# Spreading the disease: Protest in times of pandemics

**DOI:** 10.1002/hec.4602

**Published:** 2022-09-20

**Authors:** Martin Lange, Ole Monscheuer

**Affiliations:** ^1^ ZEW Mannheim Mannheim Germany; ^2^ Department of Economics Humboldt University of Berlin Berlin Germany

**Keywords:** COVID‐19, health externalities, protests, public health

## Abstract

This study analyzes the impact of large anti‐lockdown protests on the spread of SARS‐CoV‐2 in Germany. Since protesters at such large gatherings are very mobile and largely neglect SARS‐CoV‐2 containment strategies, they may contribute to the regional transmission of the coronavirus. Employing novel data on bus connections of travel companies specialized in driving protesters to these gatherings, and exploiting the timing of two large‐scale demonstrations in November 2020, we estimate the causal impact of these protests on the spread of SARS‐CoV‐2 using an event study framework. Our findings imply sizable increases in infection rates in protesters' origin regions after these demonstrations. A month after the protests, treated areas face a relative increase in infection rates up to 35% compared to non‐treated areas. Our results shed light on public health consequences of behavior that ignores potential externalities for the society during a pandemic.

## 1 INTRODUCTION

The trade‐off between individual freedom and societal externalities creates conflicts in many dimensions of society. Examples include the externalities of pollution or those of individual risk‐taking such as speeding or smoking (e.g., Kvasnicka et al., 2018; van Benthem, 2015). During the COVID‐19 pandemic, this trade‐off is highly visible and pressing. While many public health measures, for example, on social distancing, restrict individual freedom, they aim at minimizing health costs—both for the individual and the society. However, the effectiveness of such public health measures depends crucially on the support within the population.

This support appears to be closely linked to social or civic capital. A growing literature has shown that social capital is negatively associated with the spread of SARS‐CoV‐2 (e.g., Bartscher et al., 2021, show this relationship for several European countries during the first months of the pandemic). The theoretical mechanism behind this relationship is argued to be that social capital affects the acceptance of public health measures and mitigation strategies against COVID‐19, since individuals in regions with lower social capital may feel socially less responsible or have lower trust in public institutions and information regarding the potential health risks associated with COVID‐19.^1^

A lower sense of responsibility for own actions and misperceptions about COVID‐19 may turn into fierce opposition to governmentally enforced containment strategies. One of the most visible forms of such opposition to general or specific COVID‐19 mitigation policies, such as lockdowns, mask‐wearing, or vaccination mandates, could be observed at anti‐lockdown protests in many countries (e.g., The Economist, 2020). During the second infection wave in Germany, for instance, these protests have evolved to mass‐gatherings to which protesters traveled through the entire country. Given the immense mobility of thousands of protesters and the lack of enforceable mitigation strategies at such events, these protests may have played a role in the inter‐regional transmission of SARS‐CoV‐2. Accordingly, anti‐lockdown protests may have contributed to the link between social capital and infection rates.

This paper documents that protests against policies designed to curb infections with SARS‐CoV‐2, indeed contribute significantly to regional disparities in infection rates. Specifically, we estimate the causal impact of two large‐scale anti‐lockdown demonstrations in November 2020 on the spread of the novel coronavirus in Germany. For identification, we exploit the particularity that an alliance of bus companies has specialized in transporting anti‐lockdown protesters to rallies across Germany. Using web‐scraped data on all possible points of departure offered by this alliance allows us to identify the home regions of protesters. This information is used in an event study framework where we compare the development of infection rates in regions with and without such bus stops before and after the demonstrations.

While we observe parallel trends in SARS‐CoV‐2 infection rates prior to the events, our results show a significant increase in infection rates in home areas of protesters after the demonstrations. The effects are most pronounced in regions where bus stops exist even in small towns with fewer than 20,000 residents. This finding is in line with the interpretation that regions with the highest demand for transportation to the demonstrations see the highest increases in SARS‐CoV‐2 infections after the protests. We estimate that those areas faced a 35.9% higher infection rate by the end of 2020.

We perform a large number of sensitivity checks and address several threats to identification. Most importantly, we investigate whether alternative mechanisms drive our results, and flexibly extend our model to allow counties with different characteristics to experience different infection dynamics over time. First, we extend the model by pre‐determined regional proxies of social capital and the acceptance of non‐pharmaceutical interventions (NPI) to disentangle general, potentially time‐varying effects of social capital and the additional effect of anti‐lockdown protests. Allowing these county‐level characteristics to have differential effects on our outcome variables over time does not render our results insignificant. Instead, controlling for these measures of acceptance of mitigation strategies reduces our estimated effect sizes by about one third. We conclude that anti‐lockdown protests indeed have an additional effect on infection rates. Second, we employ web‐scraped data on bus stops offered by a large commercial bus travel operator, and perform a placebo test on commercial bus stops. We find that our results are not driven by differences in connectedness that may be reflected in having a bus connection to the protest sites. Third, we address the concern that protest participation could be correlated with other omitted variables that may affect infection dynamics. We flexibly control for county‐level characteristics that may favor outbreaks of SARS‐CoV‐2, such as nursing home capacities, population density, or GDP per capita. Results are robust to these extensions. Fourth, our results do not depend on states that see a particularly high increase in infection rates or share a border with highly affected neighboring countries. Finally, we show that our results are qualitatively unchanged when altering specifications, outcome definitions, or treatment definitions.

Our study contributes to three strands of literature. First, this study contributes to a growing body of work that is interested in the relationship between social capital and public health. Previous work documented a strong link between social capital and health outcomes (D'Hombres et al., 2009; Hollard & Sene, 2016; Rocco et al., 2013). Recent studies extend this literature by showing that areas with higher social capital see a slower spread of SARS‐CoV‐2 and larger reductions in individual mobility than do regions with lower social capital (Bargain & Aminjonov, 2020; Barrios et al., 2021; Bartscher et al., 2021; Borgonovi et al., 2021; Brodeur et al., 2021; Durante et al., 2021). Our study contributes to this literature by directly analyzing the consequences of large gatherings of individuals that neglect SARS‐CoV‐2 containment strategies. Thereby, this article demonstrates the importance of two key aspects of the link between social capital and the spread of SARS‐CoV‐2: adverse collective behavior in conjunction with high mobility.

Second, this paper relates to the rapidly growing economics' COVID‐19 literature that analyzes factors that affect the spread of SARS‐CoV‐2. Specifically, it contributes to those papers that focus on the role of large‐scale events or gatherings for the spread of SARS‐CoV‐2. Recent studies document that sports events that took place at a time when mitigation strategies were still absent (or not strictly enforced), increased infections and deaths associated with COVID‐19 (Ahammer et al., 2020; Breidenbach & Mitze, 2021; Carlin et al., 2021; Dave, McNichols et al., 2020). In terms of political rallies and protests, evidence on public health consequences is mixed. While Dave, Friedson et al. (2021) do not find evidence that SARS‐CoV‐2 infections were affected by a Donald Trump rally in Tulsa County in the United States, Bernheim et al. (2020), who investigate a series of Donald Trump rallies, find that these events indeed facilitated the spread of SARS‐CoV‐2 and increased deaths related to COVID‐19. Investigating SARS‐CoV‐2 infections around the time of the riot at the U.S. Capitol on January 6, 2020, Dave, McNichols et al. (2021) supply evidence that counties, from which many individuals came to participate in the riot, witnessed an increase in SARS‐CoV‐2 growth rates. Studies on the impact of Black Lives Matter protests—at which participants largely complied with coronavirus mitigation strategies—find no or only a marginal effect on SARS‐CoV‐2 infections (Dave, Friedson et al., 2020; Neyman & Dalsey, 2020). Our study contributes to this literature by analyzing SARS‐CoV‐2 infections in the home regions of protesters after two large‐scale political protests organized by COVID‐19 skeptics and at which participants deliberately neglected hygiene rules.

Finally, our study relates to the literature that is concerned with public health consequences from externalities of individual behavior—for instance on presenteeism, that is, working sick (Pichler et al., 2021; Pichler & Ziebarth, 2017), speeding (Ang et al., 2020; van Benthem, 2015) or smoking (Kvasnicka et al., 2018; Ward et al., 2013). We contribute to this literature by documenting that individual opposition to adopt containment strategies in an ongoing pandemic imposes negative externalities to others and the public health sector in general.

This paper continues as follows. In the next section, we provide background information about the course of the COVID‐19 pandemic as well as on anti‐lockdown protests in Germany. Section 3 introduces the empirical strategy and presents our causal event study estimates on the effect of anti‐lockdown protest on the spread of SARS‐CoV‐2. In Section 4, we investigate potential alternative mechanisms, other threats to identification, and study the heterogeneity of the main effects. Finally, we discuss the magnitude of our estimates and conclude in Section 5.

## 2 BACKGROUND

### 2.1 COVID‐19 in Germany

In Germany, the Robert Koch Institut (RKI) advises disease and epidemic control and collects official statistics about SARS‐CoV‐2 cases and deaths related to COVID‐19. Our analysis uses RKI data on the daily number of infections at the county level^2^ reported from January 1, 2020 to December 23, 2020.^3^ In most of our empirical analysis, we use the so‐called seven‐days‐incidence rate. It reports new SARS‐CoV‐2 cases in the last seven days per 100,000 residents in a county. Because the median incubation time (the time it takes before an individual develops symptoms of COVID‐19) is 5–6 days, with a maximum of 14 days, and because of the time it takes to get tested and receive results, it may be seven to 20 days before a SARS‐CoV‐2 infection is reported to a public health office (Lauer et al., 2020).^4^ SARS‐CoV‐2 infection statistics, thus, inhere a substantial time lag, and this has to be kept in mind when interpreting these figures.

In 2020, the RKI officially reported 1,752,015 cases and 35,373 deaths associated with COVID‐19. Beginning in late January 2020, SARS‐CoV‐2 started to disperse throughout Germany as a result of several regional clusters. On March 13, German states started to impose restrictions on public life. By mid‐April, the number of cases had fallen substantially, and many restrictions were eased (Robert Koch Institut, 2020c). During the summer, the number of infections was rather low. By late August, infection numbers started to rise again, and the second infection wave was underway in mid‐October. In response, a partial lockdown was imposed on November 2. Restaurants and bars were closed, as were cultural and leisure facilities. There was also an urgent appeal to the population to keep all personal contact to a minimum (Bundesregierung, 2020). The infection numbers stabilized at a high level. On November 25 and December 15, the chancellor and the state premiers agreed to extend the partial lockdown, deciding on slightly more stringent coronavirus protection measures but relaxing the rules about gathering over the Christmas holidays.^5^

**FIGURE 1:**
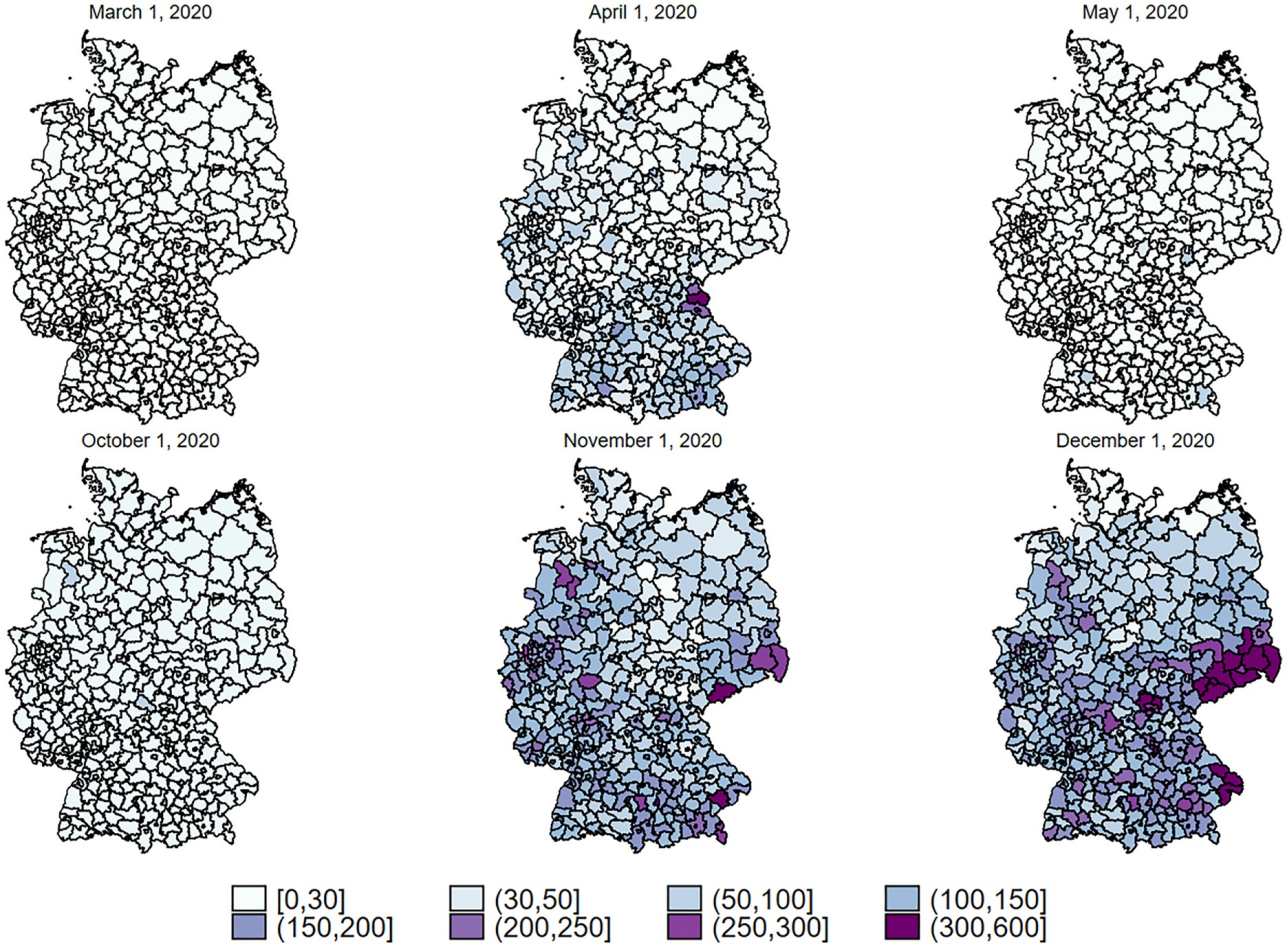
SARS‐CoV‐2 incidence rates across Germany over time. The figure shows the seven‐days‐incidence rate for counties at different points in time. The seven‐days‐incidence rate indicates the number of SARS‐CoV‐2 cases in the last seven days per 100,000 residents

While SARS‐CoV‐2 outbreaks during the first wave were mostly driven by distinct clusters, the second wave saw infections more evenly distributed across Germany. Increasingly, infections were caused by diffuse transmission, not able to be traced back to the root of the infection (Robert Koch Institut, 2020a). However, there still existed great regional heterogeneity, especially during the partial lockdown in November. Figure 1 illustrates this heterogeneity by reporting the seven‐days‐incidence rate for counties at different points in time. Figure 1 visualizes that North German regions had substantially lower infection rates than South German regions during both infection waves. The maps also show that most East German states have had very low infection rates during the first wave. This tendency shifted dramatically during the second wave when southern East German states such as Saxony reported the highest infection rates in Germany.

### 2.2 Anti‐lockdown protests

Governmental COVID‐19 containment measures spurred protests in many countries around the world. Protesters sought to display their discontent with government regulation, usually deeming the measures of restricting individual freedom as too severe. These so‐called anti‐lockdown protests became popular platforms for COVID‐19 skeptics to express their views about belittling the threat of COVID‐19 (e.g., Nachtwey et al., 2020; The Economist, 2020). In Germany, the main force behind such protests has been a group called “Querdenken” (lateral thinking), which was initially based in Stuttgart, but soon also organized rallies in other cities. Protesters at such rallies have been a diverse group of people that were only loosely united around their opposition to public containment policies (Morris & Beck, 2020; Soldt, 2020).

Since summer 2020, the Querdenken group was able to mobilize its supporters all around Germany to take part in its demonstrations through a logistical relationship with Honk for Hope (HfH). HfH was originally a group of small bus operators who opposed lockdown measures, but then started to operate as a regular COVID‐19 protest travel agency (Machowecz, 2020; Soldt, 2020). This attention to logistics has enabled the Querdenken movement (and related organizations) to mobilize very large numbers of supporters all around Germany to take part in its demonstrations.

One of its largest demonstrations took place in November 2020. On November 7, massive numbers of protesters congregated at a Querdenken demonstration in Leipzig's city center. The police spoke of 20,000 participants; a research group at the University of Leipzig estimated a crowd size as large as 45,000 (Forschungsgruppe Durchgezählt, 2020). Rioting, violent attacks on the press and police, and ongoing violations of public health regulations continued through the day. In the aftermath, the city of Leipzig, the police, and the Ministry of the Interior were criticized nationwide (Mitteldeutscher Rundfunk, 2020; Morris & Beck, 2020).

A few days later on November 18, Querdenken and related groups announced a blockade of the federal parliament to prevent a vote on an amendment to the Infection Protection Act. Tensions ran high as protesters attempted to reach the Reichstag Building, where the parliament was in session to discuss the law. The police estimated that about 10,000 protesters joined the rally (Der Polizeipräsident in Berlin, 2020). On the same day, verbally aggressive individuals entered the federal parliament, allegedly by invitation of the populist, far‐right party AfD, and threatened members of the parliament (Pechtold et al., 2020).

Querdenken demonstrations (and anti‐lockdown protests organized by related groups) have been subject to public debate especially at times of high seven‐days‐incidence rates, since participants of these protests collectively neglect hygiene regulations—e.g., requirements for social distancing or wearing face masks. If any participants have been infected with SARS‐CoV‐2, these demonstrations are primed to become superspreader events. Given the travel support provided by the bus lines, it is easy for protesters to travel within Germany to attend the demonstrations and likely that they are spreading the novel coronavirus during or after the rally. This is particularly true for demonstrations in November 2020 when infection rates were very high.

## 3 EMPIRICAL ANALYSIS

We analyze the causal effect of the two large anti‐lockdown protests in November 2020 on the infection rates in protesters' home counties. To estimate the causal effect of the anti‐lockdown demonstrations in November 2020 on SARS‐CoV‐2 infections in the origin counties of the protesters, we use the locations of HfH bus stops and employ a dynamic difference‐in‐differences design.

### 3.1 Empirical strategy

We focus on the two rallies in November for two reasons. First, infections in November were already on a high level, making it very likely that infectious protesters attended the rallies. Second, a nationwide partial lockdown was in place. Thus, governmental restrictions applied to all regions within Germany, increasing the comparability of counties in terms of regulatory responses in order to curb infections.^6^ We expect the demonstration in Leipzig on November 7 to have the greatest impact on infections. It was by far the largest demonstration in November, and the risk of infections carried by its participants to their origin counties was particularly high. Moreover, media reported SARS‐CoV‐2 cases among participants after the demonstration (e.g., Tagesspiegel, 2020). The rally in Berlin on November 18 attracted about 10,000 protesters and also may have spread infections in protesters' home regions.

For identifying protesters home counties, we web‐scraped data on possible cities of departure from the website of Honk for Hope/Kaden‐Reisen, from which protesters could book a trip to the anti‐lockdown demonstrations in Leipzig and Berlin.^7^ Exactly how many buses and passengers travel through Germany on a demonstration weekend is not known, but news articles speak of hundreds of buses at the demonstration in Leipzig on November 7 (Machowecz, 2020; Soldt, 2020).

**FIGURE 2:**
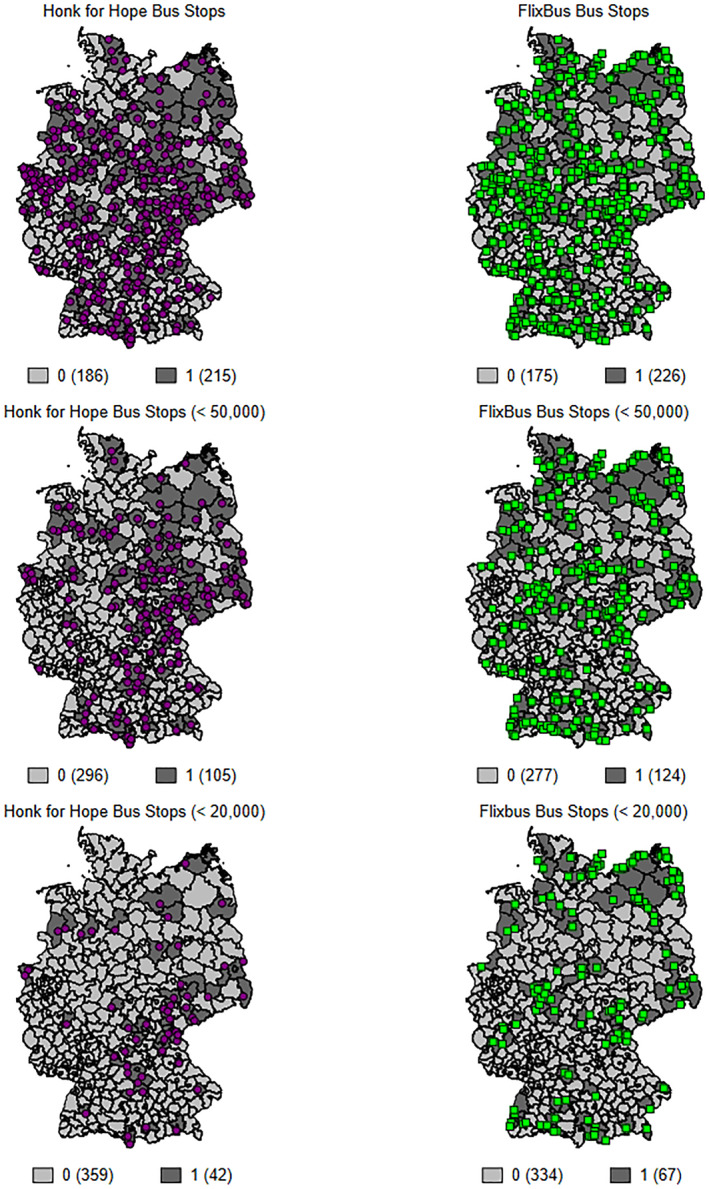
Distribution of Honk for Hope and FlixBus bus stops. The figure shows the regional distribution of Honk for Hope and FlixBus bus stops across Germany. The dark shaded areas correspond to the treated counties according to the three treatment definitions employed: having a bus stop in general, having a bus stop in cities with fewer than 50,000 inhabitants, having a bus stop in a city with fewer than 20,000 inhabitants

HfH offers bus stops in most large cities, indicating that it takes advantage, at least in part, of a general network of bus companies. However, when comparing the regional distribution of the HfH bus stops with those of FlixBus, a major bus company in Europe with significant coverage in Germany, it becomes clear that HfH is concentrated in particular regions in Germany. This is even more evident when looking at its bus stops in small and medium‐sized cities. Figure 2 shows the distribution of HfH and FlixBus bus stops in cities in general (top row), and in cities with fewer than 50,000 residents (middle row) and those with 20,000 or fewer residents (bottom row). Overall, about 54% of all German counties have a HfH bus stop (dark gray). Twenty‐six percent of the counties have a bus stop in a city with fewer than 50,000 inhabitants. About 10% of German counties have a bus stop in a town with fewer than 20,000 inhabitants.

It is likely that the HfH bus stops in smaller cities exist largely to meet the high demand for transportation to COVID‐19 demonstrations. If that is the case, protest participation in these areas may be especially high relative to local population size. Consequently, we expect the causal effect of the November demonstrations to be particularly large in counties with HfH bus stops in small cities. Hence, we run separate analyses for three different treatment definitions, where treated counties have (1) HfH bus stops in general, (2) HfH bus stops in cities with fewer than 50,000 inhabitants, or (3) HfH bus stops in cities with fewer than 20,000 inhabitants. We restrict our sample to rural counties, since counties with only one large city cannot be treated according to the second and third definition.^8^

Since infections take roughly 10 days to appear in the data of the RKI, November 18 is the first day when infections from the Leipzig demonstration may be detected. That is the date where we center the event study. Generally, we would expect infection rates to grow slowly after November 17. We restrict our sample to observations up to December 23, since infection statistics after that date becomes less reliable due to the Christmas holidays. Accordingly, we can observe the outcomes 35 days after the event. We drop observations prior to October 14, which is 35 days before the event.^9^

We run variants of the following model:

(1) $$\begin{array}{ccc} Y_{ct} & = & \gamma_{c}+\gamma_{t}+\sum_{j=-35}^{-2}\pi_{j}D\left\{Bus_{c}\right\}*D\left\{t=j\right\}+\sum_{j=0}^{35}\phi_{j}D\left\{Bus_{c}\right\}*D\left\{t=j\right\}\\ & & +\sum_{j=-35}^{-2}\alpha_{j}X_{c}*D\left\{t=j\right\}+\sum_{j=0}^{35}\beta_{j}X_{c}*D\left\{t=j\right\}+\varepsilon_{ct} \end{array}$$

Our main outcome *Y*_*ct*_ is the daily seven‐days‐incidence rate. *γ*_*c*_ and *γ*_*t*_ are county and time fixed effects. Our variables of interest are *D*{*Bus*_*c*_} ∗ *D*{*t* = *j*}. *D*{*Bus*_*c*_} is a treatment indicator that is equal to one if a county has HfH bus stops and zero otherwise (according to one of the three treatment definitions). The treatment variable is interacted with the event‐study dummy variables that are equal to one if an observation is *j* days from November 18. *π*_*j*_ capture pre‐treatment trends in *Y*_*ct*_, and *ϕ*_*j*_ are post‐treatment coefficients. They identify the differential growth in daily seven‐days‐incidence rates in the aftermath of the demonstrations in counties with HfH bus stops.

*X*_*c*_ includes control variables at the county level, which are also interacted with relative time dummy variables. In our baseline specification, *X*_*c*_ includes the value of the seven‐days‐incidence rate on November 7, allowing for different development in SARS‐CoV‐2 cases depending on the initial level of infections. Standard errors are clustered at the county level.

The main assumption to identify a causal effect is that the outcome *Y*_*ct*_ in counties with HfH bus stops would have developed parallel to those in other counties, had protesters not been present at the demonstrations in Leipzig and Berlin. When plotting the raw seven‐days‐incidence rates separately for treated and control counties in Figure A3 in the online appendix, trends are indeed very parallel throughout the second infection wave up to about 10 days after the demonstration in Leipzig. During the first infection wave in early 2020 when large demonstrations were not common, trends are also very similar—especially when defining treatment and control counties based on having a bus stop in cities smaller than 20,000 inhabitants. For the other definitions, the infection levels are slightly lower among treated counties during the first wave. The event study approach allows us to investigate the parallel trends assumption more directly through the visualization of pre‐treatment trends *π*_*j*_.

### 3.2 Main results

**FIGURE 3:**
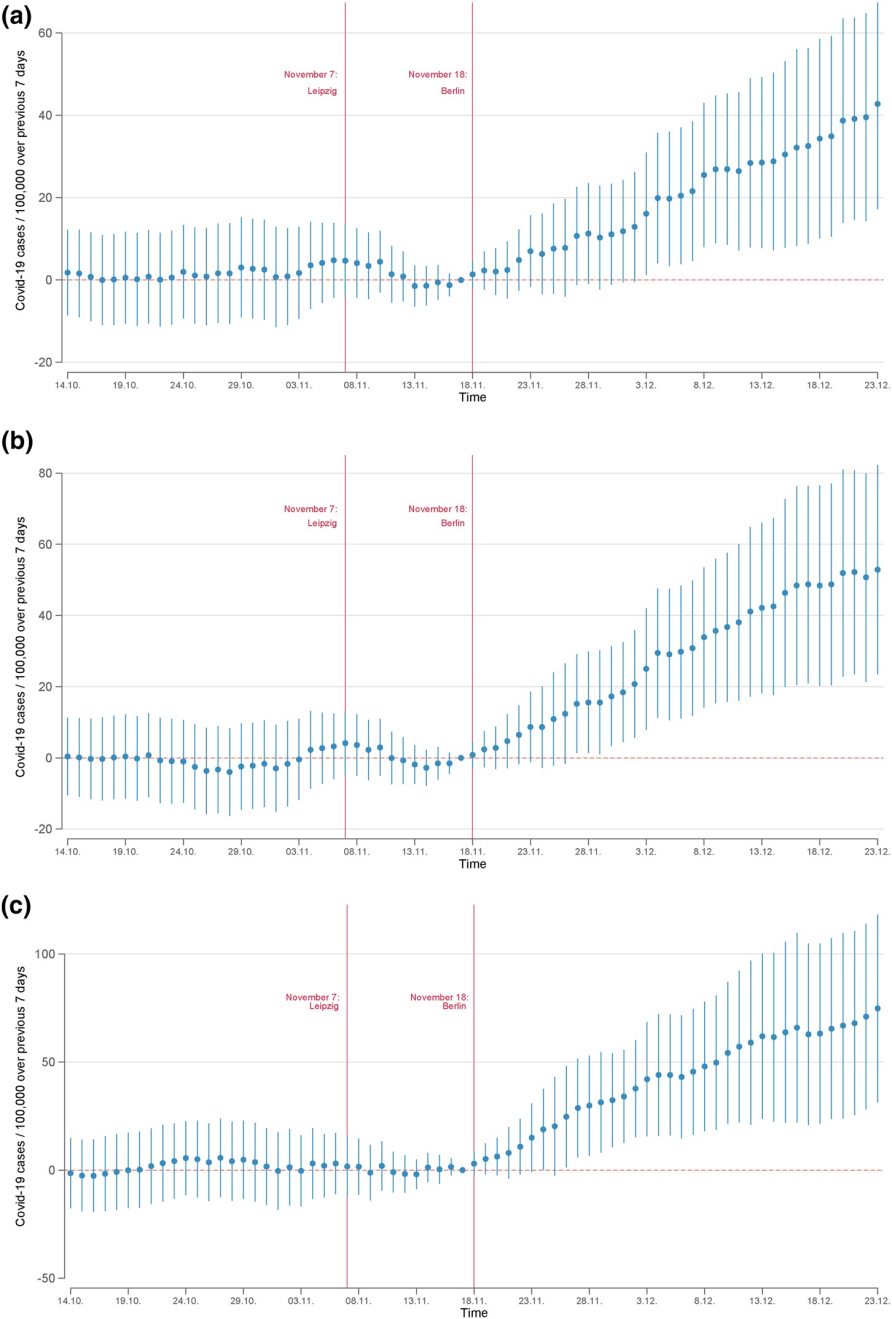
Event study results—anti‐lockdown protests and seven‐days‐incidence rates. The figures plot the event study coefficients and their 95% confidence intervals on the effect of the Querdenken demonstrations in Leipzig and Berlin on the seven‐days‐incidence rates in German counties. The treated group are counties with Honk for Hope bus stops in general, in cities with fewer than 50,000 inhabitants, or in cities with fewer than 20,000 inhabitants. The sample is restricted to rural counties and a 35‐day window around November 18. All models control for county and day fixed effects, as well as interactions between days and the incidence rate on November 7. (a) Treated: Honk for hope stops. (b) Treated: Honk for hope stops (cities smaller than 50,000). (c) Treated: Honk for hope stops (cities smaller than 20,000)

Figure 3 presents our event study estimates. The three graphs plot the coefficients *π*_*j*_ and *ϕ*_*j*_ from regression model (1) and their 95% confidence intervals for the three definitions of our treatment. The corresponding regression results can be seen in Table A2 in the online appendix.

For all three definitions of treated counties, the figures show a very parallel trend in infection rates over the entire pre‐treatment period. Conditional on the control variables, counties with HfH bus stops did not have significantly different growth rates in SARS‐CoV‐2 cases during the second wave before the demonstrations in Leipzig and Berlin. This parallel trend is observable up to about November 20. Afterward, there is a clear break, and the coefficients in all three models increase until the end of our observation window (December 23). About 2 weeks after the demonstration in Leipzig and 1 week after the demonstration in Berlin, counties that include cities that have a HfH bus stop experience higher SARS‐CoV‐2 incidence rates. These higher rates become statistically significant at a 5% level by the end of November. On December 23, counties with HfH bus stops in small towns (<20,000 inhabitants) have seven‐days‐incidence rates about 75 cases higher per 100,000 residents than counties without such bus stops. Given an average seven‐days‐incidence rate of 284 in treated counties, this effect corresponds to a 35.9% increase in the seven‐days‐incidence rate on December 23 ((100/(284 − 75)) ∗ 75).

The general picture is similar across the three definitions of our treatment groups. However, there are notable differences in the effect size that support our causal interpretation. In particular, the increases in the incidence rates are much higher and more significant among counties with HfH bus stops in small towns, compared to the effect when we employ the alternative treatment definitions. In fact, the effect size monotonically increases as the treatment definition becomes stricter. This aligns with the idea that HfH bus stops in small towns reflect demand for travel to anti‐lockdown demonstrations and, therefore, indicate a larger relative number of protesters, or at least a large number of protesters that uses bus transportation to travel to the events.

Our estimates identify diverging trends in infection rates between treated and control areas. Note that the estimated coefficients appear to increase linearly over time, as opposed to exponentially. One possible explanation for this linear divergence may lie in the *R*‐value, that is, the average number of people that one infected person will pass on the virus to. According to the RKI, the average *R*‐value in Germany was around 1 in November and December 2020 (Robert Koch Institut, 2020b, 2020d)—presumable due to the partial lockdown. Since the *R*‐value among the group of protesters is probably above average due to their low acceptance of mitigation strategies, infections contracted at the demonstrations should lead to an exponential growth in infection rates among this group relative to the control group. However, they will also spread the virus among other people in their county that take mitigation strategies more seriously and therefore stop the infection chains (e.g., because of isolation or quarantine). These transmissions may then slow down infection dynamics and prevent infection rates to diverge exponentially within the time span considered in this analysis.

## 4 ROBUSTNESS AND ADDITIONAL RESULTS

### 4.1 Alternative mechanisms

Our event study results identify diverging trends in infection rates between counties that did or did not offer bus travels to two large anti‐lockdown demonstrations in Leipzig and Berlin in November 2020. Given the timing of the diverging trends in seven‐days incidence rates between treated and control counties, our interpretation of the results is that the protests have contributed causally to the spread of SARS‐CoV‐2. This could have happened because protesters have contracted SARS‐CoV‐2 during the (mainly outdoor) protests or during the bus rides at which NPIs were unlikely to be strictly enforced.^10^ ^,^ ^11^

The interpretation of our event study results relies on the assumption that incidence rates in treated and control counties would have developed in parallel over time in absence of the events. HfH bus stops are of course not distributed at random over the country, but reflect the demand for protesting against COVID‐19 related policies. This demand may correlate with other factors, such as social capital, or demographic and economic factors that could impact infection dynamics. Hence, even though we observe parallel trends prior to the events and control for time‐constant differences across counties, there may still exist threats to our causal interpretation, if treated and control counties developed differently in late November and December for other reasons than the anti‐lockdown rallies. In this section, we investigate potential alternative mechanisms and other threats to identification. In particular, we address several potential concerns and alternative explanations by extending the set of control variables in our baseline Equation (1), that is, we extend *X*_*c*_ in $\sum_{j=-35}^{-2}\alpha_{j}X_{c}*D\left\{t=j\right\}$ and $\sum_{j=0}^{35}\beta_{j}X_{c}*D\left\{t=j\right\}$, to allow counties with different characteristics to face different infection dynamics.

Since we observe the largest effects for counties with HfH bus stops in small towns with fewer than 20,000 inhabitants, we focus our robustness section on this treatment definition and its effect on the seven‐days‐incidence rate.^12^

#### 4.1.1 Social capital, COVID‐19 skepticism, or demonstration participation?

One alternative interpretation of our results could be that the demand for bus travels to anti‐lockdown protests approximates counties' social capital or acceptance of NPIs. In fact, people who are willing to protest against mitigation measures may be more likely to ignore NPIs in everyday life at home. While the time‐constant differences across counties are controlled for in our main specification, counties with low social capital or pronounced COVID‐19 skepticism may still face different infection dynamics in November and December 2020, such that results may be partly driven by differences in social capital or COVID‐19 skepticism.

We address this issue by introducing three proxy variables on social capital and the acceptance of COVID‐19 mitigation strategies, and extend our baseline model by adding interactions between quartile dummies of these variables and time dummies to allow counties with different levels of social capital/COVID‐19 skepticism to have different infection rates over time.^13^ First, we follow the literature and use electoral turnout as a proxy for social capital. Specifically, we use the county‐level voter turnout at the last nation‐wide election before the outbreak of the COVID‐19 pandemic, the election of the European Parliament in 2019 (see, e.g., Bartscher et al., 2021).

The estimates in column (1) of Table A3 in the online appendix illustrate that the main results are robust to allowing counties above the 25th, 50th, and 75th percentile of the social capital distribution to experience different dynamics in the seven‐days incidence rates. As before, trends are parallel before the demonstrations, and *ϕ*_*j*_ increase after November 20 (*j* = 2). The effect size becomes slightly smaller compared to the main specification (e.g., 71.6 instead of 74.8 for December 23/*j* = 35). The event study coefficients of treated counties from this specification are plotted in Figure A5a in the online appendix. The graph largely resembles the main results in Figure 3.

To capture county differences more directly connected to the acceptance of COVID‐19 containment policies, we extend *X*_*c*_ with quartiles of two proxy variables for COVID‐19 skepticism: First, we use data on the vote shares of the “Alternative für Deutschland” (AfD) from the election of the European Parliament in 2019.^14^ We focus on the AfD as it is the largest political party that acknowledges the concerns of COVID‐19 skeptics, and regularly downplays the risks of COVID‐19.^15^ Moreover, since a large part of its supporters are voters that oppose the “established parties”, their vote share should reflect the regional distribution of low trust in political institutions. The pre‐determined AfD vote shares should therefore capture the political dimension of skepticism towards COVID‐19 containment policies. As a second proxy for COVID‐19 skepticism, we use county‐level data on measles vaccination rates. Specifically, we employ data on the share of children born in 2014 who received their first measles vaccination at age 15 months. All federal states recommend that children receive their first vaccination against measles by this age. Regional variation in measles vaccination rates at this age may therefore reflect mistrust in public health that correlates with opposition to containment measures against COVID‐19.

The regression models in columns (2) and (3) of Table A3 in the online appendix include interactions between time fixed effects and AfD vote share and measles vaccination rate quartile dummies, respectively. The results are robust to both extensions. As before, trends in both models are parallel up to about November 20, and diverge until the end of our observation window, such that counties with HfH bus stops experience higher incidence rates. The effect size is almost unchanged when flexibly controlling for measles vaccination rates in column (3), but it decreases somewhat when allowing counties in the different quartiles of the AfD vote share distribution to have different infection dynamics in column (2) (e.g., 44.1 instead of 74.8 for December 23/*j* = 35). An explanation for the decreasing effect size could be that the AfD vote share may not only have the advantage of reflecting differences in the acceptance of COVID‐19 containment policies across counties, but it may be also related to protest participation. Hence, it could partly take up identifying variation in the estimation of the main effect.

Finally, the regression model in column (4) includes all controls used in this section, that is, interactions between time fixed effects and quartile dummies of voter turnout, AfD vote shares, and measles vaccination rates. Again, results are robust to the inclusion of all control variables, and effect sizes are similar to the those in column (2).

Overall, our effect remains positive and significant for all three proxies of social capital and COVID‐19 skepticism, such that behavioral differences in high versus low social capital counties seem not to drive our results. We conclude that the estimated excess infections in treated counties predominantly arise due to protest participation.

#### 4.1.2 HfH bus stops as a proxy for mobility?

Another threat to the causal interpretation of our results could be that our treatment definition may reflect the connectedness of counties more generally, independent of the distribution of anti‐lockdown protesters. More specifically, HfH bus stops may simply resemble the regional distribution of bus connections. If bus stops correlate with the increase in SARS‐CoV‐2 infections, this omitted variable will bias our findings.

The map of bus stops of HfH and FlixBus in Figure 2 in Section 3.1 already suggests that the HfH network does not perfectly correspond to the FlixBus network, which should reflect the genuine demand for mobility. Nonetheless, in order to empirically elucidate whether HfH bus stops simply reveal the connectedness of the regions, we extend *X*_*c*_ of Equation (1) by a dummy variable indicating whether a county has a FlixBus stop in a small town with fewer than 20,000 inhabitants.^16^ The results in column (1) of Table A4 in the online appendix show that our main results remain almost unchanged when allowing counties with FlixBus bus stops to have different infection rates over time. In particular, we see that the pre‐treatment coefficients *π*_*j*_ are close to zero and insignificant, while *ϕ*_*j*_ again increase after November 20 (*j* = 2) through to the end of our observation window. The effect size decreases only slightly (e.g., 72.9 instead of 74.8 for December 23/*j* = 35). Figure A5b in the online appendix displays the coefficients *ϕ*_*j*_ for this specification.

In addition, Figure A5c in the online appendix plots the event study coefficients *α*_*j*_ and *β*_*j*_ for the FlixBus dummy variables from this specification. They can be interpreted as a placebo test, that is, visualizing whether infection rates develop similarly to our main results, when another set of counties with bus connections would have been treated. FlixBus stops, however, appear very differently related to the development in the seven‐days‐incidence rates than do HfH bus stops. This finding supports the argument that our treatment definition identifies regions with a relatively large number anti‐lockdown protesters, and not more or less connected regions.

#### 4.1.3 Other determinants of infection dynamics

If counties with HfH bus stops are, by coincidence, counties that favor the spread of SARS‐CoV‐2 for other reasons than protest participation, this may affect our results. Generally, this threat is rather unlikely, given the parallel trends in our main specification prior to the demonstrations. In this subsection, we still investigate this issue by extending *X*_*c*_ by different factors that may favor the spread of SARS‐CoV‐2.

First, we flexibly control for different variables that aim to approximate structural factors associated with faster outbreaks of SARS‐CoV‐2. In particular, we add quartile dummies of the counties' population density, nursing home capacities, and share of asylum applicants to *X*_*c*_. Indeed, the estimates in column (2) of Table A4 in the online appendix show that our main results are very robust to the inclusion of the additional controls variables. The effect size decreases only slightly compared to the previous specification (e.g., 67.3 instead of 74.8 for December 23/*j* = 35). The event‐study coefficients *ϕ*_*j*_ of this specification are plotted in Figure A5d in the online appendix.

Second, we investigate whether counties' economic conditions affect our results. For this, we add quartile dummies for the local unemployment rate and GDP per capita to *X*_*c*_ in column (3) of Table A4 in the online appendix. The results are highly robust when allowing for counties with dissimilar economic power to see differing growth in incidence rates. The results are very similar to the previous models, both with respect to the effect size and the statistical significance. This is also visible when plotting coefficients *ϕ*_*j*_ of this specification in Figure A5e in the online appendix.

In column (4) of Table A4 in the online appendix, we add the full set of control variables to *X*_*c*_, including our variables that were used to control for differences in social capital, connectedness, infection risk, and economic conditions. Again, results are qualitatively robust to this specification, and the effect size of *ϕ*_*j*_ is somewhat smaller than in the main specification (e.g., 46.5 instead of 74.8 for December 23/*j* = 35). Figure A5f in the online appendix plots the event study coefficients of the treated counties from the full specification. Again, the pattern in this graph is fairly similar to the main results in Figure 3.

#### 4.1.4 Region‐specific dynamics

Another potential concern with regard to the causal interpretation of our results might be that our results could be driven by omitted factors particular to specific regions. If, for example, our results are entirely driven by counties in Saxony, our treatment effects may reflect variation in the proximity to Czech Republic, which had higher infection rates in October. One argument against this concern is that we cover some of this heterogeneity in our main specification by controlling for county fixed effects and by allowing different growth in the incidence rates depending on the initial level of the seven‐days‐incidence rate. Moreover, previous extensions of our baseline model may address this concern to some extent. To test for this more explicitly, we re‐run our baseline model and successively exclude each federal state from the sample. The results are plotted in Figures A6 and A7 in the online appendix. They show that our main results are not explained by particular federal states, for example, Saxony (Figure A7c in the online appendix) or Bavaria (Figure A6g in the online appendix), as the results look similar across the 14 graphs. This is evidence that proximity to Czech Republic with its high infection rates did not cause our results.

### 4.2 Further robustness checks

#### 4.2.1 Alternative specifications

While the pre‐trends of the event study are parallel between treated and control group, one may be concerned that these trends depend on the fact that we add an interaction between the seven‐day‐incidence rate on November 7 with time fixed effects to allow for different infection dynamics depending on the pre‐demonstration level of infections.

An argument against this concern is that the raw pre‐trends in Figure A3 in the online appendix are parallel before the two demonstrations took place. Nonetheless, we further analyze the sensitivity of our results to this specification choice in Figure A8 in the online appendix: We first run our main specification, but exclude the interactions between seven‐days‐incidence rate on November 7 and time dummies (a). We then extend the analysis by controlling more flexibly for the infection dynamics of counties before the first event. In (b), we do not impose a linear relationship but add interactions of the quartiles of the incidence rate on November 7 and time dummies. We then replace the seven‐days‐incidence rate on November 7 by the growth rate in (c) and (d), that is, incidence on November 7 divided by incidence on October 31, or the absolute growth in (e) and (f), that is, incidence on November 7 minus incidence on October 31. Results in Figure A8 in the online appendix are almost identical to our main results. Hence, the specification choice of how to control for infection dynamics before the events does not affect our results.

#### 4.2.2 Alternative outcomes

Next, we test the sensitivity of our main results with regard to the definition of our outcome variable, the seven‐days‐incidence rate. A first concern may be that absolute differences between counties may become proportionally less important over time with growth in SARS‐CoV‐2 infections among all counties. We address this issue in Table A5 in the online appendix by re‐running our main models using transformed outcome variables. Specifically, our outcome in column (1) is normalized by taking the daily mean and variance into account: $\frac{\left(Y_{ct}\;-\;\bar{Y_{t}}\right)}{\sqrt{Var\left(Y_{t}\right)}}$. In column (2), we use the logarithm of the seven‐days‐incidence rate. Results in Table A5 in the online appendix are qualitatively very similar to the main results. Most importantly, the parallel trend assumption holds for the transformed outcomes. Also, the deviations from the parallel trend are the same as for the non‐normalized variables, both in terms of the size and significance of the coefficients. This result can also be seen in Figure A5g in the online appendix, which illustrates the estimated coefficients for the normalized seven‐days‐incidence rate for counties with fewer than 20,000 inhabitants (coefficients from Table A5 in the online appendix, column (1)).

A second potential concern in this respect may be that the incidence rate depends on testing. If testing capacities change selectively across counties over time, this may bias our results. To overcome this concern, we re‐run our main specification for the seven‐days‐fatality rate, which gives the number of COVID‐19 related deaths per 100,000 inhabitants over the previous seven days. An argument to use the fatality rate is that it is more precisely measurable. Results in column (3) of Table A5 in the online appendix show that the COVID‐19 fatality trends prior to the demonstrations are parallel as the pre‐treatment coefficients *π*_*j*_ are close to zero and insignificant. After the demonstrations, the coefficients are rather noisily estimated but starting around December 8, the seven‐days‐fatality rate veers upward. The delay in this increase is to be expected given the average time it takes for higher SARS‐CoV‐2 infection rates to be reflected in COVID‐19 related deaths. Due to the rather low number of cases in our outcome variable, the effect size is small, but coefficients are significant at a 10% significance level by the end of our observation window. Counties with HfH bus stops see 1.3 more deaths per 100,000 over seven days leading up to December 23 than do counties without bus stops in small towns. In fact, this reflects an increase in the seven‐days‐fatality rate of about 33% in treated counties, given a mean value of the treated group on December 23 of 6.3 ((100/(6.3 – 1.3)) ∗ 1.3). Coefficients for this outcome are plotted in Figure A5h in the online appendix.

In a next step, we show that the results on the seven‐days‐incidence are also reflected when analyzing the effect of the November demonstrations on the cumulative number of cases per 100,000 inhabitants. Column (4) of Table A4 in the online appendix again shows very parallel trends between treated and non‐treated counties when it comes to the development of total cases per 100,000. This trend diverges after November 20, as does the trend in the seven‐days‐incidence rates. The coefficients increase until the end of our observation window. On December 23, counties with HfH bus stops in small towns (<20,000 inhabitants) have 231 more SARS‐CoV‐2 cases per 100,000 inhabitants than those without bus stops. The mean value of cases is 2001 in treated counties on December 23, implying that the demonstrations increased the total number of cases in treated counties by about 13.1% ((100/(2001 − 231)) ∗ 231).

#### 4.2.3 Alternative treatment definitions

Finally, we investigate whether our results depend on the list of HfH stops that we use to construct the treatment status of counties. As described earlier, we combine in our main analysis the points of departure to both demonstrations in Berlin and Leipzig. In Figure A5 in the online appendix, we present the results when defining treated counties by having HfH bus stops to Leipzig (i) and Berlin (j) separately. Results are very similar among the different lists of bus stops, indicating that our results do not depend on a particularity of either of the two events.

### 4.3 Heterogeneity

In this subsection, we extend our analysis by exploring the heterogeneity of our main results. In order to do so, we interact our post‐treatment dummy variables with dummy variables capturing a median split in a range of covariates, that is, we add the triple‐interaction terms *D*{*Bus*_*c*_} × *D*{*t* = *j*} × *X*_*c*_ as well as the corresponding interaction terms *X*_*c*_ × *D*{*t* = *j*} to the main specification.^17^ We analyze heterogeneity with respect to the pre‐event infection rates on November 7, 2020, turnout at the election of the European parliament in 2019 as a measure of social capital, the minimum euclidean distance to Leipzig and Berlin from the counties' geographic centers, as well as GDP per capita and population density as of 2017. In congruence with previous analyses in this section, we estimate these extended models for the treatment definition that is based on having HfH bus stops in cities with less than 20,000 residents.^18^

Figure A9 in the online appendix plots the triple‐interaction terms (i.e., the coefficients of *D*{*Bus*_*c*_} × *D*{*t* = *j*} × *X*_*c*_) in the left column, and the main effect (i.e., the coefficients of *D*{*Bus*_*c*_} × *D*{*t* = *j*}) of the same specification in the right column. The triple‐interaction terms are almost always statistically insignificant. In addition, the post‐treatment interaction terms for pre‐event infection levels (a), social capital (c), and population density (i) are close to zero. For the minimum distance to Leipzig or Berlin (e), the triple‐interaction terms increasingly turn negative over time, such that farther away counties tend to face smaller treatment effects. This finding may be rationalized by a potentially decreasing protest attendance for counties located farther away from the protest sites. Similarly, counties with above median GDP per capita appear to have smaller treatment effects. As richer areas may be able to be more effective in limiting the spread of the virus, this potential heterogeneity is well in line with epidemic models of spatial virus transmission.

## 5 DISCUSSION AND CONCLUSION

This study documents that large‐scale demonstrations, at which protesters opposed and largely neglected hygiene requirements, favor the spread of SARS‐CoV‐2. Our event‐study estimates provide causal evidence that two mass protests in November 2020 in Germany significantly increased infection rates in protesters' home regions. We identify home regions of protesters by exploiting the geography of a bus network that specialized in transporting protesters to these rallies. By tracing infection rates before and after the protests for counties with and without a bus stop of this network, we find that counties with such a bus stop report a 35.9% higher seven‐days‐incidence rate by the end of 2020.

Our estimated effects on SARS‐CoV‐2 transmission are—at first sight—substantially larger than estimates that have been reported by other studies analyzing infection dynamics after large‐scale gatherings. For instance, Ahammer et al. (2020) and Dave, McNichols et al. (2020) estimate an increase in cumulative SARS‐CoV‐2 cases around 13% from sport events that took place in the United States. These events, however, took either place at the beginning of the pandemic in March 2020 (Ahammer et al., 2020) with only few infectious people moving around, or over the summer (Dave, McNichols et al., 2020)—a time at which coronavirus transmissions are less frequent. A more comparable setting is investigated by Breidenbach and Mitze (2021). Analyzing the impact of football matches in Germany in September and October 2020, they find an 8% increase in SARS‐CoV‐2 infection rates for games with up to 10,000 spectators within 14 days after the events. Since this post‐event observation period is rather short considering that infections are subject to a substantial delay before they show up in the statistics, their estimates may built up to comparable figures estimated in this article. Note, however, that at least one of the events under study in this article has attracted up to 45,000 participants, who largely neglected SARS‐CoV‐2 containment strategies. Furthermore, the protests took place in a period with a high level of infections, increasing the probability that many infectious people attended the rallies.

A back‐of‐the‐envelope calculation suggests that the two protests analyzed in this study led to at least 15,000–21,000 additional SARS‐CoV‐2 infections by the end of 2020.^19^ These estimates underscore the health costs of mass gatherings without any (enforceable) coronavirus containment strategies. Moreover, these results highlight the clash between civil liberties and public health that most governments around the world currently face. Since the public health costs associated with this trade‐off are hard to judge for policymakers, our estimates provide some indication of their size.

While this study documents increased SARS‐CoV‐2 infections after two large‐scale anti‐lockdown protests, it is less clear how policy makers should respond to rallies at which participants neglect hygiene rules. Using a small‐scale survey on COVID‐19 mitigation strategies and beliefs, we analyze how mitigation efforts correlate with trust in institutions and beliefs about the threat posed by COVID‐19.^20^ The descriptive results suggest that effort in mitigating the risk of SARS‐CoV‐2 infections correlates positively with trust in public health authorities, the government, and scientists. In addition, believing that contracting SARS‐CoV‐2 is unlikely correlates with low effort in adapting mitigation strategies. Accordingly, policy makers should aim at fostering trust in institution and supply easy‐accessible information about the threat of COVID‐19. Furthermore, insights from social psychology imply that a more positive communication approach increases the willingness to comply with public health regulations (Petersen et al., 2022).

## CONFLICT OF INTEREST

Martin Lange and Ole Monscheuer declare that they have no known competing financial interests or personal relationships that could have appeared to influence the work reported in this paper.

## Supporting information

Supplementary Material 1

## Data Availability

The data that support the findings of this study are available from the corresponding author upon reasonable request.

## ENDNOTES

^1^
For example, a notable segment of the population denies the threat posed by—or even the existence of—the novel coronavirus SARS‐CoV‐2. According to survey data from YouGov Cambridge (2020), 13% of U.S. residents believe the coronavirus probably or definitely does not exist. Similar figures are reported for Germany and France (10%).

^2^
German counties correspond to the NUTS 3 level in the EU geocode standard and are comparable in size to U.S. counties.

^3^
We restrict our analysis up to this time for three reasons: First, data from public health offices on SARS‐CoV‐2 infections is less reliable between 24.12.2020 and early 2021 due to Christmas holidays. Moreover, new variants of the coronavirus as well as the rollout of vaccinations against SARS‐CoV‐2 started in 2021, making infection rates less comparable across counties.

^4^
For example, Mitze et al. (2020) estimate for the first infection wave that SARS‐CoV‐2 cases are on average visible in the data after about 11.7 days.

^5^
Figure A2 in the online appendix visualizes the development of SARS‐CoV‐2 infections and associated deaths in Germany in 2020.

^6^
In fact, the November lockdown represents a unique setting during the current COVID‐19 pandemic in Germany, as the federal political system has been reflected in different legislation across federal states most of the time. Moreover, analyzing the infection dynamics in late 2020 has the advantage that they are not affected by the regional particularities of the spread of new variants of the coronavirus or the rollout of vaccinations.

^7^
We downloaded this information on two demonstrations in Leipzig and Berlin offered by Honk for Hope in December 2020 and January 2021. The list of possible departures is very persistent over time, and the combination of the two lists of departures represents the nationwide network of bus stops established since August 2020. As a robustness check, we show that results are very similar when using the two lists separately.

^8^
A balance table of covariates and outcomes can be found in Panel B of Table A1 in the online appendix.

^9^
The results do not depend on the choice of the event date or the time period before or after the event (see also Figure A4 in the online appendix).

^10^
Another channel could be that protesters at these large gatherings of like‐minded may confirm and reinforce anti‐mitigation attitudes and shift behavior even more away from NPI rules after traveling back home. However, one may argue that this process already happens prior to such events on social media and messenger services, such that it is unclear whether on site events further influence belief formation.

^11^
While, in this subsection, we conduct several robustness checks to argue that our results are caused by the events, we cannot further disentangle whether infections occur during the outdoor mass gatherings or predominantly during the bus rides.

^12^
Robustness checks for the other treatment definitions are in line with the main results.

^13^
Indeed, having a HfH bus stop correlates negatively with our social capital proxies, see Panel B of Table A1 in the online appendix.

^14^
The results do not change when using AfD vote shares from the last federal election in 2017.

^15^
According to Roose (2020), two thirds of AfD supporters agree or think that it is very likely that the coronavirus is a pretext in order to suppress the people—by far the largest share compared to the supporters of other political parties in Germany.

^16^
The same results are obtained when defining the FlixBus dummy variable as counties that have FlixBus stops in general or in cities with fewer than 50,000 inhabitants.

^17^
Note that the other additional variables *D*{*Bus*_*c*_} × *X*_*c*_ and *X*_*c*_ are captured by the county fixed effects.

^18^
Results for the other treatment definitions follow the same pattern, but feature smaller magnitudes of the triple‐interaction coefficients which are almost always statistically indistinguishable from zero.

^19^
These numbers result from projecting the total number of new cases per 100,000 inhabitants from the different specifications to the actual population size in treated counties: ((231/100,000) ∗ 6,699,568) = 15,476 for treated with fewer than 20,000 inhabitants; ((83/100,000) ∗ 25,574,316) = 21,227 for treated (all). These numbers should be seen as lower bounds as our estimates are based only on rural districts. Additional infections in city districts may add to the total amount of SARS‐CoV‐2 infections that originated from transmissions from protest participants.

^20^
A detailed description of this analysis including the main findings can be found in the online appendix.
