# Supplementary material for: Spreading the disease: Protest in times of pandemics
Source: Health Econ. 2022 Sep 20:10.1002/hec.4602. Online ahead of print. doi: 10.1002/hec.4602 (PMC9538087; doi:10.1002/hec.4602)
Supplement: Supplementary file 1 — Supplementary Material 1 [file HEC-9999-0-s001.pdf]

Online Appendix

Spreading the Disease:
Protest in Times of Pandemics

Martin Lange[1] and Ole Monscheuer[2]

[1] *ZEW Mannheim*
[2] *Humboldt University of Berlin*

Contents

A Online Appendix **1**
A.1 Data . . . 1
A.2 Survey Evidence . . . 3
A.3 Additional Tables and Figures . . . 5

Bibliography **33**

A Online Appendix

A.1 Data

COVID-19 Statistics

Data on SARS-CoV-2 infections and associated deaths are retrieved from the Robert Koch Institut (RKI) via the RKI COVID-19 Datahub. The RKI receives the number of SARS-CoV-2 cases and the number of deaths from the public health offices of the German states which, in turn, obtain this information from their local branches. Each case has a reporting date, which is the date on which the local public health office became aware of the case and recorded it electronically.

In most of our empirical analysis, we use the so-called seven-days-incidence rate. It reports SARS-CoV-2 cases in the last seven days per 100,000 residents in a county. Using the weekly SARS-CoV-2 rates solves two problems at once. First, it alleviates the dependency on the day of the week, as some local public health offices report SARS-CoV-2 numbers on the weekend while others do not. Second, normalizing by local population makes these rates comparable across counties within Germany.

Bus Stops

We retrieved the data on possible cities of departure from the website of Honk for Hope / Kaden-Reisen. We downloaded the bookable points of departure to two demonstrations in Leipzig and Berlin offered by HfH in December 2020 and January 2021. First, we downloaded the list of bus stops for a demonstration in Leipzig that was planned to take place on December 19 (date of access on December 11, 2020). Second, we downloaded the list of departures offered to a demonstration in Berlin on March 20, 2021 (date of access on January 28, 2020). The list of possible departures is very persistent over time, but HfH offers fewer stops very close to the respective destination.

In addition to HfH bus stops, we have acquired information on the number of FlixBus bus stops at the county level via web-scraping. FlixBus is the largest commercial long-distance bus company operating in Germany. We use the presence of its bus stops to flexibly control for the demand for bus travel.

Control Variables

We use several control variables at the county level in our empirical analysis.

Data on voter turnout and the vote share for the AfD come from the Bundeswahlleiter. We retrieve this data for the last federal election in 2017 and the election for the European parliament in 2019, which was also conducted nationwide.

The data about children's measles vaccination rates comes from the *KV-Impfsurveillance* provided by vacmap (see Rieck et al., 2018, for further information). It is based on physicians' billing to the Association of Statutory Health Insurance Physicians and includes all vaccinations administered under the public health insurance scheme. We utilize information on the share of children who received their first measles vaccination at age 15 months. All federal states

recommend that children receive their first vaccination against measles by this age. The data refer to the cohort born in 2014.

We further control for regional factors associated with a faster outbreak of SARS-CoV-2. In particular, we obtained county-level data on population density from the Federal Statistical Office. It is measured as of the population per square kilometer on December 31, 2019. In addition, we use information on capacities of nursing homes per 10,000 residents and the number of asylum applicants per 1,000 residents since SARS-CoV-2 proved to spread in large-size co-living arrangements. This data is retrieved from the INKAR data base and refers to 2017. Second, we use data on the local unemployment rate and GDP per capita to control for economic differences among counties. These figures also come from INKAR and refer to the same base year.

GESIS Panel Study

In order to analyze individual compliance with mitigation recommendations we use the special COVID-19 questionnaire of the GESIS Panel Study (GESIS Panel Team, 2020). The special COVID-19 survey is conducted on a random set of the larger GESIS Panel Study and covers 3,765 respondents surveyed March 17–29, 2020. We restrict the sample to individuals who are of age 18 or above. Summary statistics on all used variables of the survey can be found in Table A6.

In order to obtain a coherent measure of individual mitigation efforts, we perform a principle component analysis by including several questions on the adoption of mitigation strategies. Specifically, we use the binary answers to the question “Which of these measures have you taken in the last seven days?”. Respondents could indicate that they adopted the following precautions: “I avoided (crowded) public places”, “I kept 1.5 meter distance to other people”, “I washed my hands longer and more often”, “I used disinfectant”, “I reduced physical meetings and contacts”, “I worn a facial mask”. In addition, we included the answer of respondents whether they would obey to a curfew.

Dummy variables about individual institutional trust were created from answers on how much individuals trust a given institution. Answers could be given on a five-point Likert scale and ranged from “Don’t trust at all” to “Entirely trust”. If respondents answered “Rather trust” or “Entirely trust” we set the trust variables equal to one and else to zero.

We also employed information about the indivduals’ beliefs on how likely it is to contract, spread, and being hospitalized by SARS-CoV-2. Specifically, we considered the answers to the following four questions: “How likely is it in your opinion that in the next two months... (1) ...you get infected with the coronavirus? (2) ...someone from your close social surroundings (family, friends, colleagues) gets infected with the coronavirus? (3) ...you need to be hospitalized in case you get infected with the coronavirus? (4) ...you get infected with the coronavirus and spread it to other people?”. Answers to these questions could be given on a seven-point Likert scale ranging from “Not at all likely” to “Absolutely likely”. If respondents indicated that the event already happened, we assigned their response to “Absolutely likely”. Moreover, we generate a dummy variable that shall capture a strong disbelief in the threats posed by COVID-19. We classify respondents as belittling the threat of COVID-19 if they answer every of the aforementioned questions with “Not at all likely” or “Very unlikely”. Three percent of the sample are classified as belittling COVID-19 risks according to this definition.

A.2 Survey Evidence

In this section, we use individual-level survey data to see whether trust in institutions and beliefs about the health risk carried by the novel coronavirus is linked to individual engagement in mitigation strategies against the spread of and contracting SARS-CoV-2.

Figure A1: Mitigation Efforts, Trust in Institutions, and Beliefs about COVID-19

Dependent Variable: Mitigation Efforts

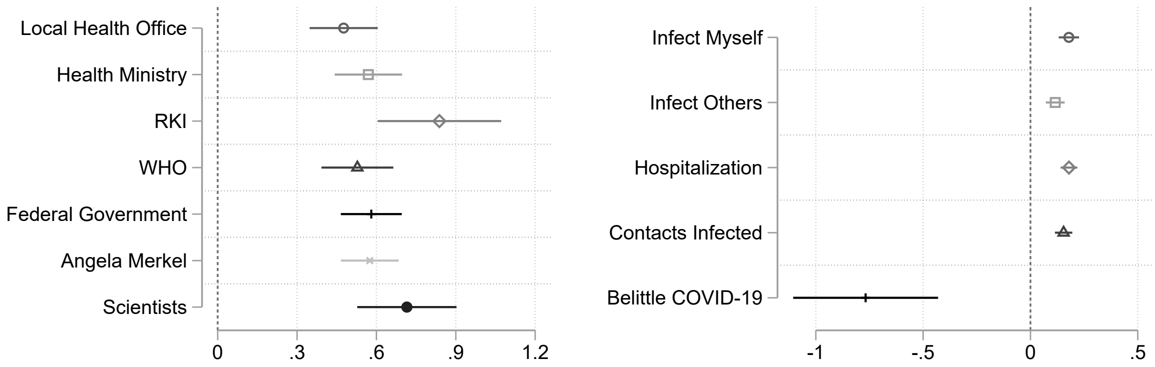

(a) Trust in Institutions (b) Beliefs about COVID-19

Notes: The subfigures plot the regression coefficients of separate OLS regressions of the principal component of individual mitigation efforts on (a) a dummy variable indicating whether a person trust in a specific institution or (b) on the belief how likely it is to contract SARS-CoV-2. Regressions include the following control variables: gender, age, education, household size, and labor force status. Heteroskedasticity robust standard errors were estimated. 95 percent confidence intervals are shown. Data comes from the GESIS Panel Study.

We exploit individual survey data from a special COVID-19 questionnaire of the GESIS Panel Study (GESIS Panel Team, 2020)—one of the few representative survey data sets on COVID-19 in Germany. The special COVID-19 survey is conducted on a random set of the larger, nationally representative GESIS Panel Study and covers 3,765 respondents surveyed March 17–29, 2020. It entails information about the precautions that a person undertakes to mitigate the spread of the coronavirus, trust in institutions, as well as individuals' assessments about the likelihood of contracting, spreading, and getting hospitalized from SARS-CoV-2.

The survey asks many question about the mitigation behavior of the respondent, e.g. whether individuals engage in reducing contacts, avoiding public places, wearing masks, etc. In order to have a single coherent measure of mitigation efforts, we generate the principle component of all mitigation strategies that have been asked in the survey. Next, we perform several separate regressions in which we regress the principal component of mitigation behavior on different trust indicators and assessments on how likely it is that the respondent or other persons contract SARS-CoV-2. In each of these regressions, we control for a set of basic socioeconomic variables including gender, age, education, household size, and labor force status. Figure A1 plots the coefficients of interest, while the full regression outputs can be found in Tables A7 and A8. In addition, we provide summary statistics of the employed variables in Table A6.

Panel (a) of Figure A1 documents that individual mitigation efforts correlate significantly with trust in different institutions that have been authorities in informing the public about COVID-19 and giving advice on how to behave during the pandemic. The point estimates refer to the respective trust measure for each regression on the principle component of mitigation efforts. The plot clearly visualizes that individuals, who have a high degree of trust in the

respective institution are significantly more likely to adapt mitigation strategies. Especially those who trust the RKI and have a high confidence in science in general are substantially more likely to engage in mitigating behavior.

Panel (b) shows how respondents' assessments on the likelihood to contract SARS-CoV-2 corresponds to the adoption of mitigation strategies. Individuals, who believe that it is likely that they self or direct contacts may be infected with the coronavirus, comply with a higher propensity with mitigation recommendations. The same result is visible for individuals, who believe that they may be hospitalized or that they may infect other people, if they contract SARS-CoV-2. Individuals who belittle the threat posed by COVID-19 are statistically significantly less likely to adopt recommended mitigation behavior.

A.3 Additional Tables and Figures

Table A1: Summary Statistics and Treatment Balancing

**Panel A: Summary Statistics**	Mean	Median	SD	Min	Max	Counties
7 Day Incidence Rate on Nov 1, 2020	119.57	115.58	58.27	14.98	334.81	401
7 Day Incidence Rate on Dec 1, 2020	141.57	131.91	77.04	8.45	499.97	401
7 Day Fatality Rate on Nov 1, 2020	1.58	1.10	1.84	0.00	13.06	401
7 Day Fatality Rate on Dec 1, 2020	3.88	2.74	4.91	0.00	56.12	401
Counties with Honk for Hope Bus Stops	0.54	1.00	0.50	0.00	1.00	401
in Cities below 50,000 Residents	0.26	0.00	0.44	0.00	1.00	401
in Cities below 20,000 Residents	0.10	0.00	0.31	0.00	1.00	401
Counties with FlixBus Bus Stops	0.56	1.00	0.50	0.00	1.00	401
in Cities below 50,000 Residents	0.31	0.00	0.46	0.00	1.00	401
in Cities below 20,000 Residents	0.17	0.00	0.37	0.00	1.00	401
Measles Vaccination Rate at Age 15-Months	88.95	90.30	4.92	72.40	97.50	401
Vote Share of AfD at EU Parliament Election 2019	11.47	9.56	5.53	3.99	32.21	401
Electoral Turnout at EU Parliament Election 2019	60.59	60.62	4.82	47.62	74.38	401
Population Density	536.86	201.00	709.70	36.00	4777.00	401
Unemployment Rate	5.36	5.00	2.41	1.50	14.00	401
GDP per Capita in EUR 1,000	37.09	33.10	16.05	16.40	172.40	401
Nursing Home Capacity per 10,000 Residents	113.19	108.50	28.86	51.40	235.80	401
Asylum Applicants per 1,000 Residents	5.39	5.10	2.57	0.00	31.90	401

	Total		Cities<50,000		Cities<20,000	
**Panel B: Treatment Balancing**	Diff.	p-Value	Diff.	p-Value	Diff.	p-Value
7 Day Incidence Rate on Nov 1, 2020	-4.83	0.46	4.22	0.65	-14.72	0.03**
7 Day Incidence Rate on Dec 1, 2020	6.33	0.48	38.47	0.00***	6.64	0.49
7 Day Fatality Rate on Nov 1, 2020	0.10	0.65	0.96	0.00***	0.04	0.86
7 Day Fatality Rate on Dec 1, 2020	0.00	1.00	1.91	0.01**	0.20	0.73
Measles Vaccination Rate at Age 15-Months	-0.63	0.30	-0.97	0.26	-0.71	0.27
Vote Share of AfD at EU Parliament Election 2019	2.80	0.00***	4.11	0.00***	3.72	0.00***
Electoral Turnout at EU Parliament Election 2019	-1.83	0.00***	-1.76	0.02**	-1.79	0.00***
Population Density	-31.36	0.16	-109.53	0.00***	-86.40	0.00***
Unemployment Rate	1.20	0.00***	0.47	0.16	1.06	0.00***
GDP per Capita in EUR 1,000	1.10	0.28	-0.49	0.74	-0.40	0.71
Nursing Home Capacity per 10,000 Residents	9.28	0.01***	5.80	0.22	9.46	0.01***
Asylum Applicants per 1,000 Residents	0.39	0.08*	0.26	0.41	0.19	0.41

Notes: Panel A reports summary statistics on the main outcome, treatment, and control variables for all 401 counties in Germany. Panel B shows level differences in the means of outcome and control variables between counties that have at least one Honk for Hope bus stop and those that do not for our estimation sample of rural counties. Statistical significance in mean differences is indicated by asterisks according to: * $p < 0.10$, ** $p < 0.05$, *** $p < 0.01$.

Table A2: Event Study Results: Seven-Days-Incidence Rate

**Dependent Variable: Seven-Days-Incidence Rate**			
	Bus Stops (all)	*Bus Stops (<50,0000)*	*Bus Stops (<20,0000)*
	(1)	(2)	(3)
$D\{Bus_c = 1\} * D\{j = -35\}$	1.817	0.405	-1.403
	(5.276)	(5.536)	(8.239)
$D\{Bus_c = 1\} * D\{j = -34\}$	1.580	0.135	-2.542
	(5.383)	(5.635)	(8.395)
$D\{Bus_c = 1\} * D\{j = -33\}$	0.764	-0.257	-2.630
	(5.469)	(5.763)	(8.505)
$D\{Bus_c = 1\} * D\{j = -32\}$	-0.004	-0.287	-1.663
	(5.553)	(5.911)	(8.797)
$D\{Bus_c = 1\} * D\{j = -31\}$	0.121	0.110	-0.832
	(5.627)	(5.968)	(8.867)
$D\{Bus_c = 1\} * D\{j = -30\}$	0.567	0.395	-0.084
	(5.651)	(6.002)	(8.860)
$D\{Bus_c = 1\} * D\{j = -29\}$	0.178	-0.167	0.217
	(5.736)	(6.026)	(9.007)
$D\{Bus_c = 1\} * D\{j = -28\}$	0.808	0.756	1.844
	(5.760)	(6.001)	(8.863)
$D\{Bus_c = 1\} * D\{j = -27\}$	0.080	-0.718	3.212
	(5.785)	(6.103)	(9.031)
$D\{Bus_c = 1\} * D\{j = -26\}$	0.581	-0.936	4.191
	(5.789)	(6.055)	(8.828)
$D\{Bus_c = 1\} * D\{j = -25\}$	1.981	-0.986	5.559
	(5.762)	(5.902)	(8.641)
$D\{Bus_c = 1\} * D\{j = -24\}$	1.116	-2.511	5.047
	(5.926)	(6.101)	(9.013)
$D\{Bus_c = 1\} * D\{j = -23\}$	0.830	-3.645	3.636
	(5.996)	(6.155)	(9.097)
$D\{Bus_c = 1\} * D\{j = -22\}$	1.632	-3.276	5.700
	(6.122)	(6.216)	(9.216)
$D\{Bus_c = 1\} * D\{j = -21\}$	1.601	-3.972	4.140
	(6.206)	(6.257)	(9.303)
$D\{Bus_c = 1\} * D\{j = -20\}$	3.041	-2.416	4.815
	(6.185)	(6.175)	(9.196)
$D\{Bus_c = 1\} * D\{j = -19\}$	2.734	-2.197	3.715
	(6.130)	(6.151)	(9.249)
$D\{Bus_c = 1\} * D\{j = -18\}$	2.520	-1.632	1.684

Continued on next page

	(6.172)	(6.169)	(9.000)
$D\{Bus_c = 1\} * D\{j = -17\}$	0.708	-2.933	-0.360
	(6.153)	(6.196)	(9.111)
$D\{Bus_c = 1\} * D\{j = -16\}$	0.877	-1.660	1.353
	(5.984)	(6.096)	(8.982)
$D\{Bus_c = 1\} * D\{j = -15\}$	1.702	-0.426	-0.340
	(5.690)	(5.804)	(8.351)
$D\{Bus_c = 1\} * D\{j = -14\}$	3.573	2.276	3.068
	(5.369)	(5.559)	(8.309)
$D\{Bus_c = 1\} * D\{j = -13\}$	4.164	2.759	2.017
	(4.929)	(5.064)	(7.407)
$D\{Bus_c = 1\} * D\{j = -12\}$	4.793	3.243	3.077
	(4.621)	(4.682)	(7.187)
$D\{Bus_c = 1\} * D\{j = -11\}$	4.695	4.153	1.695
	(4.471)	(4.571)	(6.951)
$D\{Bus_c = 1\} * D\{j = -10\}$	4.108	3.627	1.577
	(4.290)	(4.395)	(6.595)
$D\{Bus_c = 1\} * D\{j = -9\}$	3.430	2.273	-1.181
	(4.103)	(4.308)	(6.519)
$D\{Bus_c = 1\} * D\{j = -8\}$	4.432	2.946	1.952
	(3.839)	(4.061)	(5.789)
$D\{Bus_c = 1\} * D\{j = -7\}$	1.411	-0.053	-0.947
	(3.490)	(3.747)	(4.772)
$D\{Bus_c = 1\} * D\{j = -6\}$	0.855	-0.698	-1.752
	(3.069)	(3.344)	(4.383)
$D\{Bus_c = 1\} * D\{j = -5\}$	-1.448	-1.847	-1.916
	(2.525)	(2.759)	(3.480)
$D\{Bus_c = 1\} * D\{j = -4\}$	-1.395	-2.765	1.240
	(2.394)	(2.559)	(3.429)
$D\{Bus_c = 1\} * D\{j = -3\}$	-0.570	-1.525	0.341
	(2.125)	(2.317)	(3.394)
$D\{Bus_c = 1\} * D\{j = -2\}$	-1.244	-1.520	1.501
	(1.399)	(1.501)	(1.979)
$D\{Bus_c = 1\} * D\{j = 0\}$	1.371	0.837	2.984
	(1.683)	(1.885)	(2.770)
$D\{Bus_c = 1\} * D\{j = 1\}$	2.316	2.422	5.183
	(2.380)	(2.576)	(3.678)
$D\{Bus_c = 1\} * D\{j = 2\}$	2.050	2.835	6.295
	(2.876)	(3.040)	(4.392)
$D\{Bus_c = 1\} * D\{j = 3\}$	2.444	4.726	7.984

Continued on next page

	(3.531)	(3.849)	(6.049)
$D\{Bus_c = 1\} * D\{j = 4\}$	4.844	6.494	10.816*
	(3.769)	(4.175)	(6.507)
$D\{Bus_c = 1\} * D\{j = 5\}$	6.998	8.700*	14.999*
	(4.387)	(5.019)	(8.090)
$D\{Bus_c = 1\} * D\{j = 6\}$	6.323	8.679	18.829**
	(4.967)	(5.816)	(9.561)
$D\{Bus_c = 1\} * D\{j = 7\}$	7.613	10.927*	20.287*
	(5.567)	(6.613)	(11.576)
$D\{Bus_c = 1\} * D\{j = 8\}$	7.808	12.410*	24.723**
	(6.016)	(7.162)	(11.901)
$D\{Bus_c = 1\} * D\{j = 9\}$	10.709*	15.224**	28.727**
	(6.062)	(7.033)	(11.591)
$D\{Bus_c = 1\} * D\{j = 10\}$	11.277*	15.604**	29.875**
	(6.187)	(7.215)	(11.741)
$D\{Bus_c = 1\} * D\{j = 11\}$	10.302	15.595**	31.371***
	(6.390)	(7.395)	(11.796)
$D\{Bus_c = 1\} * D\{j = 12\}$	11.098*	17.304**	32.377***
	(6.221)	(7.086)	(10.993)
$D\{Bus_c = 1\} * D\{j = 13\}$	11.855*	18.459***	34.063***
	(6.317)	(7.114)	(10.922)
$D\{Bus_c = 1\} * D\{j = 14\}$	12.922*	20.785***	37.731***
	(6.777)	(7.659)	(11.388)
$D\{Bus_c = 1\} * D\{j = 15\}$	16.107**	24.973***	42.070***
	(7.556)	(8.644)	(13.415)
$D\{Bus_c = 1\} * D\{j = 16\}$	19.890**	29.445***	44.053***
	(8.049)	(9.203)	(14.255)
$D\{Bus_c = 1\} * D\{j = 17\}$	19.752**	29.058***	44.020***
	(8.285)	(9.345)	(14.181)
$D\{Bus_c = 1\} * D\{j = 18\}$	20.471**	29.780***	43.103***
	(8.426)	(9.462)	(14.440)
$D\{Bus_c = 1\} * D\{j = 19\}$	21.572**	30.797***	45.546***
	(8.587)	(9.659)	(14.796)
$D\{Bus_c = 1\} * D\{j = 20\}$	25.494***	33.868***	47.997***
	(8.888)	(9.991)	(15.221)
$D\{Bus_c = 1\} * D\{j = 21\}$	26.897***	35.669***	49.741***
	(9.097)	(10.288)	(15.754)
$D\{Bus_c = 1\} * D\{j = 22\}$	26.913***	36.737***	54.245***
	(9.335)	(10.622)	(16.663)
$D\{Bus_c = 1\} * D\{j = 23\}$	26.449***	38.047***	57.151***

Continued on next page

	(9.772)	(11.178)	(17.813)
$D\{Bus_c = 1\} * D\{j = 24\}$	28.436***	41.065***	59.008***
	(10.441)	(12.088)	(19.281)
$D\{Bus_c = 1\} * D\{j = 25\}$	28.542***	42.129***	61.945***
	(10.539)	(12.163)	(19.400)
$D\{Bus_c = 1\} * D\{j = 26\}$	28.814***	42.533***	61.547***
	(10.968)	(12.618)	(19.849)
$D\{Bus_c = 1\} * D\{j = 27\}$	30.493***	46.335***	63.771***
	(11.504)	(13.411)	(21.245)
$D\{Bus_c = 1\} * D\{j = 28\}$	32.150***	48.419***	65.911***
	(12.133)	(14.174)	(22.324)
$D\{Bus_c = 1\} * D\{j = 29\}$	32.550***	48.739***	62.856***
	(12.081)	(14.072)	(21.295)
$D\{Bus_c = 1\} * D\{j = 30\}$	34.325***	48.398***	63.200***
	(12.320)	(14.302)	(21.170)
$D\{Bus_c = 1\} * D\{j = 31\}$	34.910***	48.749***	65.445***
	(12.395)	(14.384)	(21.253)
$D\{Bus_c = 1\} * D\{j = 32\}$	38.713***	51.916***	66.896***
	(12.628)	(14.790)	(21.801)
$D\{Bus_c = 1\} * D\{j = 33\}$	39.147***	52.194***	68.025***
	(12.470)	(14.577)	(21.642)
$D\{Bus_c = 1\} * D\{j = 34\}$	39.530***	50.721***	71.049***
	(12.832)	(14.902)	(21.800)
$D\{Bus_c = 1\} * D\{j = 35\}$	42.770***	52.873***	74.835***
	(12.967)	(14.923)	(22.034)
County & Time FE	Yes	Yes	Yes
7.11. Incidence * Time FE	Yes	Yes	Yes
Observations	20945	20945	20945
Adj. R-Squared	0.699	0.704	0.704

Notes: The table reports event study estimates on the effect of the Querdenken demonstrations in Leipzig and Berlin on the seven-days-incidence rates in German counties. The treated group are counties with any Honk for Hope bus stops (column (1)), with bus stops in cities with fewer than 50,000 inhabitants (column (2)), or bus stops in cities with fewer than 20,000 inhabitants (columns (3)). The sample is restricted to rural counties and a 35-day window around November 18. All models control for county and day fixed effects, as well as interactions between days and the incidence rate on November 7. Statistical significance: *** p<0.01, ** p<0.05, * p<0.1.

Table A3: Robustness: Additional Control Variables

	Dependent Variable: Seven-Days-Incidence Rate			
	Voter Turnout	*AfD Voter Share*	*Measles Vacc. Rate*	*All*
	(1)	(2)	(3)	(4)
$D\{Bus_c = 1\} * D\{j = -35\}$	-0.466	0.868	-1.404	0.762
	(8.432)	(7.885)	(8.247)	(7.847)
$D\{Bus_c = 1\} * D\{j = -34\}$	-1.756	0.128	-2.573	-0.167
	(8.628)	(8.027)	(8.390)	(8.034)
$D\{Bus_c = 1\} * D\{j = -33\}$	-1.919	0.420	-2.729	0.008
	(8.750)	(8.072)	(8.507)	(8.108)
$D\{Bus_c = 1\} * D\{j = -32\}$	-0.957	1.397	-1.774	0.950
	(9.060)	(8.254)	(8.808)	(8.317)
$D\{Bus_c = 1\} * D\{j = -31\}$	-0.262	2.199	-0.979	1.618
	(9.148)	(8.321)	(8.886)	(8.407)
$D\{Bus_c = 1\} * D\{j = -30\}$	0.481	3.368	-0.174	2.788
	(9.165)	(8.313)	(8.870)	(8.417)
$D\{Bus_c = 1\} * D\{j = -29\}$	0.661	3.657	0.110	2.953
	(9.369)	(8.417)	(9.018)	(8.596)
$D\{Bus_c = 1\} * D\{j = -28\}$	2.233	5.626	1.740	4.900
	(9.258)	(8.297)	(8.870)	(8.510)
$D\{Bus_c = 1\} * D\{j = -27\}$	3.876	7.270	3.155	6.836
	(9.385)	(8.411)	(9.038)	(8.571)
$D\{Bus_c = 1\} * D\{j = -26\}$	4.908	8.478	4.126	8.105
	(9.265)	(8.122)	(8.815)	(8.369)
$D\{Bus_c = 1\} * D\{j = -25\}$	6.167	9.963	5.436	9.407
	(9.003)	(8.014)	(8.670)	(8.239)
$D\{Bus_c = 1\} * D\{j = -24\}$	5.434	9.934	4.915	9.100
	(9.343)	(8.425)	(9.028)	(8.578)
$D\{Bus_c = 1\} * D\{j = -23\}$	3.843	9.155	3.537	8.231
	(9.424)	(8.402)	(9.110)	(8.550)
$D\{Bus_c = 1\} * D\{j = -22\}$	6.022	11.596	5.530	10.692
	(9.523)	(8.595)	(9.210)	(8.675)
$D\{Bus_c = 1\} * D\{j = -21\}$	4.382	9.617	3.911	8.625
	(9.611)	(8.790)	(9.300)	(8.893)
$D\{Bus_c = 1\} * D\{j = -20\}$	4.805	9.861	4.557	8.631
	(9.493)	(8.741)	(9.220)	(8.895)
$D\{Bus_c = 1\} * D\{j = -19\}$	3.852	8.641	3.466	7.458
	(9.465)	(8.847)	(9.257)	(8.900)
$D\{Bus_c = 1\} * D\{j = -18\}$	1.959	6.600	1.518	5.583

Continued on next page

	(9.255)	(8.567)	(8.997)	(8.659)
$D\{Bus_c = 1\} * D\{j = -17\}$	0.054	4.444	-0.538	3.586
	(9.422)	(8.592)	(9.086)	(8.709)
$D\{Bus_c = 1\} * D\{j = -16\}$	2.042	5.401	1.193	4.786
	(9.222)	(8.568)	(8.949)	(8.631)
$D\{Bus_c = 1\} * D\{j = -15\}$	0.280	3.025	-0.447	2.340
	(8.574)	(7.922)	(8.342)	(8.002)
$D\{Bus_c = 1\} * D\{j = -14\}$	3.765	5.763	3.071	5.189
	(8.497)	(7.821)	(8.287)	(7.864)
$D\{Bus_c = 1\} * D\{j = -13\}$	2.553	4.033	1.934	3.357
	(7.560)	(6.992)	(7.357)	(7.018)
$D\{Bus_c = 1\} * D\{j = -12\}$	3.377	4.356	2.987	3.581
	(7.366)	(6.855)	(7.163)	(6.895)
$D\{Bus_c = 1\} * D\{j = -11\}$	2.008	2.969	1.558	2.282
	(7.156)	(6.653)	(6.926)	(6.669)
$D\{Bus_c = 1\} * D\{j = -10\}$	1.861	2.912	1.597	2.419
	(6.769)	(6.314)	(6.594)	(6.380)
$D\{Bus_c = 1\} * D\{j = -9\}$	-0.820	0.221	-1.134	-0.073
	(6.751)	(6.334)	(6.483)	(6.467)
$D\{Bus_c = 1\} * D\{j = -8\}$	2.244	3.413	2.032	3.224
	(6.014)	(5.809)	(5.751)	(5.967)
$D\{Bus_c = 1\} * D\{j = -7\}$	-0.767	1.600	-0.868	1.367
	(4.936)	(4.780)	(4.741)	(4.876)
$D\{Bus_c = 1\} * D\{j = -6\}$	-1.351	1.114	-1.533	1.328
	(4.553)	(4.157)	(4.349)	(4.260)
$D\{Bus_c = 1\} * D\{j = -5\}$	-1.307	0.922	-1.676	1.469
	(3.574)	(3.189)	(3.458)	(3.252)
$D\{Bus_c = 1\} * D\{j = -4\}$	1.639	3.994	1.487	4.377
	(3.507)	(3.141)	(3.442)	(3.257)
$D\{Bus_c = 1\} * D\{j = -3\}$	0.954	2.281	0.524	2.865
	(3.442)	(3.235)	(3.385)	(3.326)
$D\{Bus_c = 1\} * D\{j = -2\}$	1.883	2.560	1.632	2.909
	(2.026)	(1.876)	(1.980)	(1.915)
$D\{Bus_c = 1\} * D\{j = 0\}$	2.475	3.042	2.873	2.584
	(2.757)	(2.792)	(2.794)	(2.801)
$D\{Bus_c = 1\} * D\{j = 1\}$	4.353	4.644	5.053	3.951
	(3.671)	(3.727)	(3.656)	(3.691)
$D\{Bus_c = 1\} * D\{j = 2\}$	5.185	4.486	6.115	3.592
	(4.389)	(4.541)	(4.344)	(4.495)
$D\{Bus_c = 1\} * D\{j = 3\}$	6.579	4.488	7.779	3.614

Continued on next page

	(6.180)	(5.989)	(6.008)	(6.098)
$D\{Bus_c = 1\} * D\{j = 4\}$	8.847	7.247	10.655	5.923
	(6.635)	(6.302)	(6.468)	(6.429)
$D\{Bus_c = 1\} * D\{j = 5\}$	12.786	10.615	14.859*	9.266
	(8.421)	(7.579)	(8.036)	(7.959)
$D\{Bus_c = 1\} * D\{j = 6\}$	16.556*	14.409	18.729*	13.112
	(9.978)	(9.074)	(9.530)	(9.566)
$D\{Bus_c = 1\} * D\{j = 7\}$	17.880	14.099	20.236*	12.828
	(12.166)	(10.592)	(11.432)	(11.215)
$D\{Bus_c = 1\} * D\{j = 8\}$	21.974*	16.852	24.615**	15.465
	(12.499)	(10.736)	(11.739)	(11.337)
$D\{Bus_c = 1\} * D\{j = 9\}$	25.338**	20.698**	28.643**	18.891*
	(12.089)	(10.311)	(11.459)	(10.850)
$D\{Bus_c = 1\} * D\{j = 10\}$	26.689**	22.010**	29.801**	20.479*
	(12.122)	(10.409)	(11.615)	(10.859)
$D\{Bus_c = 1\} * D\{j = 11\}$	28.355**	22.785**	31.318***	21.544**
	(12.232)	(10.472)	(11.625)	(10.892)
$D\{Bus_c = 1\} * D\{j = 12\}$	29.701***	23.010**	32.208***	21.998**
	(11.251)	(9.636)	(10.812)	(9.869)
$D\{Bus_c = 1\} * D\{j = 13\}$	31.941***	23.318**	33.894***	22.896**
	(11.112)	(9.366)	(10.690)	(9.462)
$D\{Bus_c = 1\} * D\{j = 14\}$	35.352***	25.371***	37.614***	25.065***
	(11.600)	(9.472)	(11.078)	(9.521)
$D\{Bus_c = 1\} * D\{j = 15\}$	39.561***	27.715**	41.920***	27.354**
	(13.520)	(11.179)	(13.020)	(11.087)
$D\{Bus_c = 1\} * D\{j = 16\}$	42.173***	27.871**	43.955***	28.262**
	(14.532)	(11.979)	(13.817)	(11.953)
$D\{Bus_c = 1\} * D\{j = 17\}$	42.070***	27.553**	43.830***	27.859**
	(14.524)	(12.052)	(13.724)	(12.092)
$D\{Bus_c = 1\} * D\{j = 18\}$	41.097***	26.183**	42.862***	26.463**
	(14.794)	(12.355)	(13.956)	(12.383)
$D\{Bus_c = 1\} * D\{j = 19\}$	43.208***	27.902**	45.322***	27.900**
	(15.187)	(12.546)	(14.283)	(12.514)
$D\{Bus_c = 1\} * D\{j = 20\}$	44.921***	29.429**	47.753***	28.923**
	(15.650)	(12.855)	(14.680)	(12.818)
$D\{Bus_c = 1\} * D\{j = 21\}$	47.008***	30.630**	49.407***	30.530**
	(16.094)	(13.270)	(15.190)	(13.190)
$D\{Bus_c = 1\} * D\{j = 22\}$	52.103***	34.796**	53.795***	35.296**
	(17.045)	(13.881)	(16.099)	(13.819)
$D\{Bus_c = 1\} * D\{j = 23\}$	54.359***	36.417**	56.653***	36.400**

Continued on next page

	(18.175)	(14.521)	(17.151)	(14.420)
$D\{Bus_c = 1\} * D\{j = 24\}$	56.316***	36.326**	58.530***	36.533**
	(19.679)	(15.760)	(18.511)	(15.497)
$D\{Bus_c = 1\} * D\{j = 25\}$	59.517***	38.985**	61.473***	39.593**
	(19.751)	(15.781)	(18.654)	(15.475)
$D\{Bus_c = 1\} * D\{j = 26\}$	59.074***	36.653**	61.056***	37.445**
	(20.129)	(16.093)	(19.104)	(15.734)
$D\{Bus_c = 1\} * D\{j = 27\}$	61.706***	38.237**	63.204***	39.555**
	(21.427)	(17.433)	(20.418)	(16.882)
$D\{Bus_c = 1\} * D\{j = 28\}$	63.436***	38.325**	65.379***	39.311**
	(22.480)	(18.206)	(21.504)	(17.621)
$D\{Bus_c = 1\} * D\{j = 29\}$	60.079***	35.427**	62.433***	36.367**
	(21.471)	(17.634)	(20.376)	(16.902)
$D\{Bus_c = 1\} * D\{j = 30\}$	60.168***	35.283**	62.650***	36.012**
	(21.245)	(17.772)	(20.337)	(17.110)
$D\{Bus_c = 1\} * D\{j = 31\}$	62.228***	37.690**	64.962***	38.418**
	(21.259)	(17.541)	(20.460)	(16.871)
$D\{Bus_c = 1\} * D\{j = 32\}$	63.452***	38.271**	66.436***	38.832**
	(21.808)	(17.752)	(20.984)	(17.062)
$D\{Bus_c = 1\} * D\{j = 33\}$	64.778***	39.742**	67.596***	40.437**
	(21.748)	(17.498)	(20.796)	(16.824)
$D\{Bus_c = 1\} * D\{j = 34\}$	67.486***	40.651**	70.633***	41.209**
	(22.137)	(17.619)	(20.912)	(17.014)
$D\{Bus_c = 1\} * D\{j = 35\}$	71.571***	44.091**	74.224***	44.874**
	(22.446)	(18.082)	(21.075)	(17.337)
County & Time FE	Yes	Yes	Yes	Yes
7.11. Incidence * Time FE	Yes	Yes	Yes	Yes
Voter Turnout * Time FE	Yes	Yes	Yes	Yes
AfD Vote Share * Time FE	No	Yes	No	Yes
Measles Vacc. Rate * Time FE	No	No	Yes	Yes
Observations	20945	20945	20945	20945
Adj. R-Squared	0.704	0.772	0.710	0.774

Notes: The table reports event study estimates on the effect of the Querdenken demonstrations in Leipzig and Berlin on the seven-days-incidence rates in German counties. The treated group are counties with Honk for Hope bus stops in cities with fewer than 20,000 inhabitants. The sample is restricted to rural counties and a 35-day window around November 18. All models control for county and day fixed effects, as well as interactions between days and the incidence rate on November 7. The different models add interactions between day fixed effects and quartile dummies of voter turnout (column (1)), AfD vote share (column(2)), and measles vaccination rates (column (3)). Column (4) includes interactions between day fixed effects and quartile dummies of all three variables. Standard errors in parentheses are clustered by county. Statistical significance: *** p<0.01, ** p<0.05, * p<0.1.

Table A4: Robustness: Additional Control Variables

	Dependent Variable: Seven-Days-Incidence Rate			
	FlixBus	*Structural Factors*	*Economic Conditions*	*All Variables*
	(1)	(2)	(3)	(4)
$D\{Bus_c = 1\} * D\{j = -35\}$	-5.890	-0.757	-1.854	-3.844
	(8.185)	(8.839)	(8.260)	(8.587)
$D\{Bus_c = 1\} * D\{j = -34\}$	-7.240	-2.188	-3.029	-5.081
	(8.415)	(9.068)	(8.446)	(8.813)
$D\{Bus_c = 1\} * D\{j = -33\}$	-7.413	-2.278	-3.051	-4.859
	(8.489)	(9.172)	(8.561)	(8.875)
$D\{Bus_c = 1\} * D\{j = -32\}$	-6.638	-1.444	-2.088	-3.865
	(8.789)	(9.437)	(8.885)	(9.132)
$D\{Bus_c = 1\} * D\{j = -31\}$	-6.079	-0.858	-1.210	-3.322
	(8.986)	(9.509)	(8.956)	(9.277)
$D\{Bus_c = 1\} * D\{j = -30\}$	-5.571	-0.075	-0.399	-2.436
	(8.994)	(9.569)	(8.961)	(9.353)
$D\{Bus_c = 1\} * D\{j = -29\}$	-5.388	0.142	-0.133	-2.382
	(9.307)	(9.796)	(9.125)	(9.626)
$D\{Bus_c = 1\} * D\{j = -28\}$	-3.654	1.811	1.422	-0.687
	(9.306)	(9.688)	(8.977)	(9.533)
$D\{Bus_c = 1\} * D\{j = -27\}$	-2.304	3.961	2.974	1.903
	(9.388)	(9.804)	(9.154)	(9.597)
$D\{Bus_c = 1\} * D\{j = -26\}$	-0.800	6.222	3.910	4.723
	(9.281)	(9.773)	(8.986)	(9.589)
$D\{Bus_c = 1\} * D\{j = -25\}$	0.633	7.403	5.129	5.966
	(9.165)	(9.798)	(8.704)	(9.542)
$D\{Bus_c = 1\} * D\{j = -24\}$	-0.189	6.783	4.526	5.495
	(9.475)	(10.361)	(9.144)	(10.023)
$D\{Bus_c = 1\} * D\{j = -23\}$	-1.358	6.012	3.062	4.812
	(9.556)	(10.250)	(9.177)	(9.869)
$D\{Bus_c = 1\} * D\{j = -22\}$	0.403	8.808	5.348	7.697
	(9.616)	(10.712)	(9.304)	(10.204)
$D\{Bus_c = 1\} * D\{j = -21\}$	-1.508	7.371	3.774	5.736
	(9.535)	(10.756)	(9.454)	(10.315)
$D\{Bus_c = 1\} * D\{j = -20\}$	-0.538	7.750	4.155	6.200
	(9.451)	(10.702)	(9.330)	(10.281)
$D\{Bus_c = 1\} * D\{j = -19\}$	-2.458	6.659	2.895	4.134
	(9.472)	(10.656)	(9.393)	(10.168)
$D\{Bus_c = 1\} * D\{j = -18\}$	-4.878	4.540	0.802	1.509
	(9.146)	(10.368)	(9.188)	(9.850)
$D\{Bus_c = 1\} * D\{j = -17\}$	-7.064	2.994	-1.263	-0.818
	(9.236)	(10.423)	(9.262)	(9.877)

Continued on next page

$D\{Bus_c = 1\} * D\{j = -16\}$	-5.248	4.448	0.322	0.723
	(8.985)	(10.454)	(9.138)	(9.738)
$D\{Bus_c = 1\} * D\{j = -15\}$	-6.742	2.144	-1.971	-2.117
	(8.465)	(9.625)	(8.435)	(8.981)
$D\{Bus_c = 1\} * D\{j = -14\}$	-3.261	4.499	1.633	0.298
	(8.271)	(9.423)	(8.393)	(8.702)
$D\{Bus_c = 1\} * D\{j = -13\}$	-3.868	3.002	0.350	-1.811
	(7.350)	(8.280)	(7.496)	(7.632)
$D\{Bus_c = 1\} * D\{j = -12\}$	-2.201	1.984	1.607	-2.644
	(7.213)	(8.016)	(7.255)	(7.532)
$D\{Bus_c = 1\} * D\{j = -11\}$	-2.315	-0.194	0.656	-3.609
	(7.063)	(7.649)	(6.961)	(7.358)
$D\{Bus_c = 1\} * D\{j = -10\}$	-1.638	-0.879	0.816	-3.296
	(6.719)	(7.213)	(6.615)	(7.018)
$D\{Bus_c = 1\} * D\{j = -9\}$	-3.227	-2.661	-1.939	-4.661
	(6.738)	(7.006)	(6.519)	(6.970)
$D\{Bus_c = 1\} * D\{j = -8\}$	0.724	0.523	1.848	-0.501
	(5.853)	(6.322)	(5.704)	(6.212)
$D\{Bus_c = 1\} * D\{j = -7\}$	-1.006	-1.613	-0.853	-0.696
	(5.007)	(5.423)	(4.633)	(5.351)
$D\{Bus_c = 1\} * D\{j = -6\}$	-1.732	-2.294	-1.557	-0.930
	(4.602)	(4.904)	(4.147)	(4.760)
$D\{Bus_c = 1\} * D\{j = -5\}$	-1.558	-1.056	-1.297	0.603
	(3.614)	(3.918)	(3.209)	(3.713)
$D\{Bus_c = 1\} * D\{j = -4\}$	0.898	2.952	1.827	3.825
	(3.608)	(3.788)	(3.149)	(3.636)
$D\{Bus_c = 1\} * D\{j = -3\}$	0.059	2.159	0.954	2.370
	(3.522)	(3.596)	(3.166)	(3.535)
$D\{Bus_c = 1\} * D\{j = -2\}$	0.841	2.768	1.892	2.966
	(2.044)	(2.121)	(1.947)	(2.166)
$D\{Bus_c = 1\} * D\{j = 0\}$	2.200	2.548	3.065	1.474
	(2.600)	(2.829)	(2.589)	(2.757)
$D\{Bus_c = 1\} * D\{j = 1\}$	4.665	4.721	5.441	2.985
	(3.596)	(3.968)	(3.419)	(3.777)
$D\{Bus_c = 1\} * D\{j = 2\}$	5.647	4.426	6.167	1.750
	(4.328)	(4.798)	(4.148)	(4.588)
$D\{Bus_c = 1\} * D\{j = 3\}$	7.811	5.148	8.371	2.418
	(6.189)	(6.301)	(5.943)	(6.162)
$D\{Bus_c = 1\} * D\{j = 4\}$	10.922*	7.662	10.954*	5.170
	(6.547)	(6.806)	(6.415)	(6.463)
$D\{Bus_c = 1\} * D\{j = 5\}$	15.909*	10.752	15.118*	8.430
	(8.295)	(8.023)	(8.253)	(7.885)
$D\{Bus_c = 1\} * D\{j = 6\}$	19.953**	13.807	18.986*	12.100
	(9.654)	(9.467)	(9.814)	(9.217)
$D\{Bus_c = 1\} * D\{j = 7\}$	22.275*	14.802	20.231*	12.622
	(11.887)	(11.008)	(12.035)	(10.915)

Continued on next page

$D\{Bus_c = 1\} * D\{j = 8\}$	26.188**	17.744	25.043**	15.101
	(12.339)	(10.857)	(12.347)	(11.035)
$D\{Bus_c = 1\} * D\{j = 9\}$	30.725**	21.731**	28.998**	19.359*
	(11.989)	(10.814)	(11.956)	(10.834)
$D\{Bus_c = 1\} * D\{j = 10\}$	31.838***	23.017**	29.847**	20.637*
	(12.080)	(11.031)	(12.083)	(10.943)
$D\{Bus_c = 1\} * D\{j = 11\}$	32.903***	24.487**	31.515***	21.610*
	(12.160)	(11.141)	(12.070)	(11.098)
$D\{Bus_c = 1\} * D\{j = 12\}$	33.243***	25.226**	32.857***	21.242**
	(11.266)	(10.647)	(11.058)	(10.343)
$D\{Bus_c = 1\} * D\{j = 13\}$	33.738***	28.166***	34.607***	22.117**
	(11.126)	(10.840)	(10.768)	(10.133)
$D\{Bus_c = 1\} * D\{j = 14\}$	37.326***	32.068***	38.481***	25.765**
	(11.392)	(11.565)	(10.982)	(10.188)
$D\{Bus_c = 1\} * D\{j = 15\}$	41.748***	35.914***	42.591***	28.219**
	(13.434)	(13.503)	(12.949)	(11.779)
$D\{Bus_c = 1\} * D\{j = 16\}$	43.386***	38.100***	44.586***	28.973**
	(14.281)	(14.464)	(13.726)	(12.621)
$D\{Bus_c = 1\} * D\{j = 17\}$	43.106***	38.189***	44.806***	29.121**
	(14.192)	(14.537)	(13.664)	(12.759)
$D\{Bus_c = 1\} * D\{j = 18\}$	42.729***	37.026**	44.208***	27.716**
	(14.394)	(14.872)	(14.045)	(13.032)
$D\{Bus_c = 1\} * D\{j = 19\}$	45.150***	40.374***	46.169***	30.290**
	(14.544)	(15.068)	(14.340)	(12.957)
$D\{Bus_c = 1\} * D\{j = 20\}$	47.874***	42.116***	48.300***	30.975**
	(15.193)	(15.604)	(14.827)	(13.449)
$D\{Bus_c = 1\} * D\{j = 21\}$	48.647***	43.078***	49.825***	29.926**
	(15.914)	(16.260)	(15.446)	(13.976)
$D\{Bus_c = 1\} * D\{j = 22\}$	52.904***	48.529***	54.255***	34.795**
	(16.621)	(17.109)	(16.190)	(14.382)
$D\{Bus_c = 1\} * D\{j = 23\}$	55.845***	49.242***	57.230***	35.236**
	(17.691)	(17.943)	(17.241)	(14.845)
$D\{Bus_c = 1\} * D\{j = 24\}$	57.128***	50.867***	58.204***	34.675**
	(19.072)	(19.429)	(18.612)	(16.195)
$D\{Bus_c = 1\} * D\{j = 25\}$	59.699***	54.499***	60.804***	37.599**
	(19.338)	(19.577)	(18.566)	(16.246)
$D\{Bus_c = 1\} * D\{j = 26\}$	58.533***	53.287***	61.216***	34.693**
	(19.966)	(20.053)	(18.701)	(16.524)
$D\{Bus_c = 1\} * D\{j = 27\}$	59.914***	54.320**	63.146***	35.006**
	(21.170)	(21.070)	(20.006)	(17.421)
$D\{Bus_c = 1\} * D\{j = 28\}$	61.976***	55.258**	65.253***	34.414*
	(22.112)	(22.065)	(20.687)	(18.207)
$D\{Bus_c = 1\} * D\{j = 29\}$	59.369***	51.981**	62.819***	31.542*
	(21.493)	(21.535)	(19.739)	(17.808)
$D\{Bus_c = 1\} * D\{j = 30\}$	58.749***	53.711**	62.978***	31.378*
	(21.541)	(21.526)	(19.676)	(18.111)

Continued on next page

$D\{Bus_c = 1\} * D\{j = 31\}$	61.515***	55.704**	66.181***	34.072*
	(21.559)	(21.509)	(19.719)	(17.786)
$D\{Bus_c = 1\} * D\{j = 32\}$	63.299***	57.110**	67.907***	35.252*
	(21.964)	(22.120)	(20.271)	(18.025)
$D\{Bus_c = 1\} * D\{j = 33\}$	65.287***	59.208***	67.960***	37.561**
	(22.034)	(22.174)	(20.279)	(17.995)
$D\{Bus_c = 1\} * D\{j = 34\}$	68.657***	61.753***	71.550***	40.130**
	(22.263)	(22.783)	(20.461)	(18.451)
$D\{Bus_c = 1\} * D\{j = 35\}$	72.871***	67.345***	75.719***	46.543**
	(22.346)	(22.962)	(20.811)	(18.683)
County & Time FE	Yes	Yes	Yes	Yes
7.11. Incidence * Time FE	Yes	Yes	Yes	Yes
FlixBus Stop * Time FE	Yes	No	No	Yes
Covid-Risk Controls * Time FE	No	Yes	No	Yes
Economic Controls * Time FE	No	No	Yes	Yes
Soc. Capital Controls * Time FE	No	No	No	Yes
Observations	20945	20945	20945	20945
Adj. R-Squared	0.705	0.708	0.710	0.776

Notes: The table reports event study estimates on the effect of the Querdenken demonstrations in Leipzig and Berlin on the seven-days-incidence rates in German counties. The treated group are counties with Honk for Hope bus stops in cities with fewer than 20,000 inhabitants. The sample is restricted to rural counties and a 35-day window around November 18. All models control for county and day fixed effects, as well as interactions between days and the incidence rate on November 7. Column (1) adds interactions between having a FlixBus stop and day fixed effects. Column (2) adds interactions between days and quartile dummies of population densisty, nursing home capacities per 10,000 residents, and rate of asylum seekers. Column (3) adds interactions between day fixed effects and quartile dummies of the unemployment rate and GDP per capita. Column (4) includes all control variables. Standard errors in parentheses are clustered by county. Statistical significance: *** p<0.01, ** p<0.05, * p<0.1.

Table A5: Event Study Results: Alternative Outcomes

	Dependent Variable:			
	Norm. Incidence	*Log Incidence*	*Seven-Days-Fatality*	*Total Cases / 100,000*
	(1)	(2)	(3)	(4)
$D\{Bus_c = 1\} * D\{j = -35\}$	-0.160	-0.113	0.134	-6.277
	(0.124)	(0.205)	(0.446)	(11.239)
$D\{Bus_c = 1\} * D\{j = -34\}$	-0.137	-0.149	0.094	-6.635
	(0.126)	(0.196)	(0.445)	(11.057)
$D\{Bus_c = 1\} * D\{j = -33\}$	-0.106	-0.140	0.085	-7.120
	(0.117)	(0.195)	(0.443)	(10.907)
$D\{Bus_c = 1\} * D\{j = -32\}$	-0.063	-0.094	0.037	-6.283
	(0.114)	(0.200)	(0.451)	(10.693)
$D\{Bus_c = 1\} * D\{j = -31\}$	-0.043	-0.060	-0.022	-6.456
	(0.119)	(0.197)	(0.455)	(10.668)
$D\{Bus_c = 1\} * D\{j = -30\}$	-0.007	-0.033	0.105	-5.718
	(0.111)	(0.195)	(0.462)	(10.481)
$D\{Bus_c = 1\} * D\{j = -29\}$	0.008	-0.019	0.044	-4.798
	(0.112)	(0.190)	(0.466)	(10.268)
$D\{Bus_c = 1\} * D\{j = -28\}$	0.072	0.031	0.173	-3.337
	(0.101)	(0.182)	(0.474)	(10.118)
$D\{Bus_c = 1\} * D\{j = -27\}$	0.093	0.070	0.182	-2.094
	(0.093)	(0.183)	(0.491)	(9.986)
$D\{Bus_c = 1\} * D\{j = -26\}$	0.103	0.094	0.332	-1.490
	(0.085)	(0.174)	(0.514)	(9.652)
$D\{Bus_c = 1\} * D\{j = -25\}$	0.128*	0.126	0.407	0.674
	(0.076)	(0.165)	(0.512)	(9.421)
$D\{Bus_c = 1\} * D\{j = -24\}$	0.124	0.107	0.430	0.264
	(0.080)	(0.169)	(0.500)	(9.528)
$D\{Bus_c = 1\} * D\{j = -23\}$	0.107	0.071	0.356	-0.232
	(0.078)	(0.167)	(0.506)	(9.620)
$D\{Bus_c = 1\} * D\{j = -22\}$	0.128	0.114	0.460	2.789
	(0.079)	(0.166)	(0.482)	(9.598)
$D\{Bus_c = 1\} * D\{j = -21\}$	0.092	0.077	0.450	2.397
	(0.076)	(0.164)	(0.507)	(9.523)
$D\{Bus_c = 1\} * D\{j = -20\}$	0.092	0.089	0.510	3.895
	(0.073)	(0.159)	(0.498)	(9.431)
$D\{Bus_c = 1\} * D\{j = -19\}$	0.084	0.066	0.557	3.030
	(0.074)	(0.158)	(0.471)	(9.389)
$D\{Bus_c = 1\} * D\{j = -18\}$	0.065	0.027	0.651	3.102

Continued on next page

	(0.074)	(0.151)	(0.455)	(9.085)
$D\{Bus_c = 1\} * D\{j = -17\}$	0.036	-0.010	0.673	0.426
	(0.075)	(0.152)	(0.482)	(9.622)
$D\{Bus_c = 1\} * D\{j = -16\}$	0.033	0.021	0.687	0.981
	(0.075)	(0.149)	(0.498)	(9.647)
$D\{Bus_c = 1\} * D\{j = -15\}$	0.019	-0.008	0.556	1.890
	(0.069)	(0.136)	(0.521)	(9.700)
$D\{Bus_c = 1\} * D\{j = -14\}$	0.046	0.051	0.485	4.499
	(0.067)	(0.135)	(0.524)	(9.924)
$D\{Bus_c = 1\} * D\{j = -13\}$	0.037	0.032	0.392	4.593
	(0.059)	(0.119)	(0.483)	(9.501)
$D\{Bus_c = 1\} * D\{j = -12\}$	0.036	0.050	0.230	4.767
	(0.060)	(0.115)	(0.475)	(9.278)
$D\{Bus_c = 1\} * D\{j = -11\}$	0.027	0.027	0.016	3.102
	(0.057)	(0.111)	(0.463)	(9.085)
$D\{Bus_c = 1\} * D\{j = -10\}$	0.029	0.025	0.052	0.337
	(0.053)	(0.105)	(0.454)	(8.990)
$D\{Bus_c = 1\} * D\{j = -9\}$	0.019	-0.020	0.000	-1.553
	(0.051)	(0.104)	(0.430)	(8.230)
$D\{Bus_c = 1\} * D\{j = -8\}$	0.041	0.031	0.303	2.282
	(0.043)	(0.092)	(0.477)	(6.859)
$D\{Bus_c = 1\} * D\{j = -7\}$	0.002	-0.015	0.358	2.125
	(0.038)	(0.076)	(0.409)	(5.919)
$D\{Bus_c = 1\} * D\{j = -6\}$	0.005	-0.028	0.319	1.424
	(0.037)	(0.070)	(0.375)	(5.043)
$D\{Bus_c = 1\} * D\{j = -5\}$	-0.002	-0.030	0.302	1.241
	(0.028)	(0.055)	(0.361)	(4.200)
$D\{Bus_c = 1\} * D\{j = -4\}$	0.017	0.020	0.251	2.851
	(0.027)	(0.054)	(0.305)	(3.563)
$D\{Bus_c = 1\} * D\{j = -3\}$	0.010	0.006	-0.033	-1.202
	(0.025)	(0.054)	(0.282)	(3.704)
$D\{Bus_c = 1\} * D\{j = -2\}$	0.019	0.024	0.114	-2.144
	(0.014)	(0.031)	(0.220)	(2.449)
$D\{Bus_c = 1\} * D\{j = 0\}$	0.033*	0.047	0.254	3.367
	(0.018)	(0.044)	(0.322)	(2.299)
$D\{Bus_c = 1\} * D\{j = 1\}$	0.044*	0.082	0.569	4.761
	(0.024)	(0.058)	(0.494)	(3.763)
$D\{Bus_c = 1\} * D\{j = 2\}$	0.044	0.099	0.490	5.550
	(0.029)	(0.068)	(0.519)	(5.330)
$D\{Bus_c = 1\} * D\{j = 3\}$	0.047	0.122	0.834	8.569

Continued on next page

	(0.038)	(0.091)	(0.544)	(7.277)
$D\{Bus_c = 1\} * D\{j = 4\}$	0.067	0.164*	0.977*	7.423
	(0.041)	(0.098)	(0.533)	(7.813)
$D\{Bus_c = 1\} * D\{j = 5\}$	0.084*	0.222*	0.736	10.474
	(0.046)	(0.118)	(0.551)	(9.471)
$D\{Bus_c = 1\} * D\{j = 6\}$	0.101*	0.275**	0.595	16.690
	(0.058)	(0.138)	(0.597)	(11.977)
$D\{Bus_c = 1\} * D\{j = 7\}$	0.083	0.287*	0.349	21.020
	(0.067)	(0.160)	(0.650)	(14.607)
$D\{Bus_c = 1\} * D\{j = 8\}$	0.106	0.338**	0.311	26.511
	(0.065)	(0.158)	(0.610)	(16.894)
$D\{Bus_c = 1\} * D\{j = 9\}$	0.145**	0.385**	0.794	31.337*
	(0.067)	(0.150)	(0.643)	(18.532)
$D\{Bus_c = 1\} * D\{j = 10\}$	0.143**	0.398***	1.015	35.563*
	(0.069)	(0.151)	(0.664)	(20.714)
$D\{Bus_c = 1\} * D\{j = 11\}$	0.149**	0.412***	1.194	35.841*
	(0.070)	(0.149)	(0.724)	(21.251)
$D\{Bus_c = 1\} * D\{j = 12\}$	0.165**	0.431***	1.461*	39.611*
	(0.067)	(0.140)	(0.767)	(22.703)
$D\{Bus_c = 1\} * D\{j = 13\}$	0.181***	0.451***	1.489**	47.425*
	(0.066)	(0.138)	(0.727)	(24.742)
$D\{Bus_c = 1\} * D\{j = 14\}$	0.221***	0.480***	1.354*	55.149**
	(0.067)	(0.137)	(0.735)	(27.116)
$D\{Bus_c = 1\} * D\{j = 15\}$	0.234***	0.509***	1.290	64.912**
	(0.076)	(0.154)	(0.806)	(30.919)
$D\{Bus_c = 1\} * D\{j = 16\}$	0.237***	0.519***	0.975	71.489**
	(0.080)	(0.160)	(0.780)	(33.491)
$D\{Bus_c = 1\} * D\{j = 17\}$	0.256***	0.510***	0.613	75.735**
	(0.079)	(0.157)	(0.688)	(35.430)
$D\{Bus_c = 1\} * D\{j = 18\}$	0.252***	0.492***	0.391	75.285**
	(0.079)	(0.157)	(0.672)	(36.443)
$D\{Bus_c = 1\} * D\{j = 19\}$	0.257***	0.515***	0.394	81.502**
	(0.079)	(0.159)	(0.688)	(38.191)
$D\{Bus_c = 1\} * D\{j = 20\}$	0.267***	0.530***	0.897	91.548**
	(0.078)	(0.159)	(0.688)	(41.068)
$D\{Bus_c = 1\} * D\{j = 21\}$	0.273***	0.537***	0.904	101.133**
	(0.075)	(0.160)	(0.731)	(44.039)
$D\{Bus_c = 1\} * D\{j = 22\}$	0.281***	0.575***	0.927	115.064**
	(0.075)	(0.167)	(0.720)	(48.400)
$D\{Bus_c = 1\} * D\{j = 23\}$	0.273***	0.587***	1.090	124.547**

Continued on next page

	(0.078)	(0.174)	(0.763)	(51.728)
$D\{Bus_c = 1\} * D\{j = 24\}$	0.263***	0.578***	1.320	130.211**
	(0.080)	(0.179)	(0.806)	(54.507)
$D\{Bus_c = 1\} * D\{j = 25\}$	0.270***	0.601***	1.652**	132.621**
	(0.079)	(0.178)	(0.830)	(55.519)
$D\{Bus_c = 1\} * D\{j = 26\}$	0.268***	0.580***	1.939**	138.109**
	(0.078)	(0.176)	(0.873)	(57.938)
$D\{Bus_c = 1\} * D\{j = 27\}$	0.259***	0.586***	1.649*	150.307**
	(0.083)	(0.185)	(0.859)	(61.332)
$D\{Bus_c = 1\} * D\{j = 28\}$	0.252***	0.588***	1.822**	161.494**
	(0.087)	(0.189)	(0.894)	(64.983)
$D\{Bus_c = 1\} * D\{j = 29\}$	0.252***	0.568***	1.941**	172.720**
	(0.086)	(0.183)	(0.868)	(68.159)
$D\{Bus_c = 1\} * D\{j = 30\}$	0.260***	0.569***	2.075**	182.451**
	(0.084)	(0.181)	(0.857)	(71.219)
$D\{Bus_c = 1\} * D\{j = 31\}$	0.270***	0.589***	1.682**	190.533**
	(0.083)	(0.181)	(0.851)	(74.485)
$D\{Bus_c = 1\} * D\{j = 32\}$	0.276***	0.598***	1.509*	194.307**
	(0.086)	(0.186)	(0.884)	(75.993)
$D\{Bus_c = 1\} * D\{j = 33\}$	0.277***	0.617***	1.320*	200.958**
	(0.085)	(0.188)	(0.740)	(78.543)
$D\{Bus_c = 1\} * D\{j = 34\}$	0.294***	0.631***	1.269*	216.119***
	(0.086)	(0.185)	(0.723)	(82.717)
$D\{Bus_c = 1\} * D\{j = 35\}$	0.304***	0.658***	1.314*	230.939***
	(0.086)	(0.186)	(0.719)	(86.583)
County & Time FE	Yes	Yes	Yes	Yes
7.11. Inc. / Fat. * Time FE	Yes	Yes	Yes	Yes
Observations	20945	20945	20945	20945
Adj. R-Squared	0.804	0.617	0.508	0.953

Notes: The table reports event study estimates on the effect of the Querdenken demonstrations in Leipzig and Berlin the spread of COVID-19 in German counties. The treated group are counties with any Honk for Hope bus stops in cities with fewer than 20,000 inhabitants. The outcome variable is the normalized seven-days-incidence rate (column (1)), log seven-days-incidence rate (column(2)), the seven-days-fatality rate (column(3)), and the total number of cases per 100,000 (column(4)). The sample is restricted to rural counties and a 35-day window around November 18. All models control for county and day fixed effects, as well as interactions between days and the incidence/fatality rate on November 7. Statistical significance: *** p<0.01, ** p<0.05, * p<0.1.

Table A6: Summary Statistics of the Special COVID-19 Questionnaire

	Mean	Median	SD	Min	Max	No. Obs.
Mitigation Efforts	-0.00	0.70	1.43	-5.07	1.61	3120
Avoid Places	0.85	1.00	0.36	0.00	1.00	3120
Keep Distance	0.80	1.00	0.40	0.00	1.00	3120
Wash Hands	0.91	1.00	0.29	0.00	1.00	3120
Use Disinfectant	0.60	1.00	0.49	0.00	1.00	3120
Reduced Contacts	0.85	1.00	0.35	0.00	1.00	3120
Wear Masks	0.04	0.00	0.19	0.00	1.00	3120
Accept Curfew	0.92	1.00	0.28	0.00	1.00	3108
Trust in Local Health Office	0.73	1.00	0.45	0.00	1.00	2862
Trust in Health Ministry	0.73	1.00	0.45	0.00	1.00	3058
Trust in RKI	0.91	1.00	0.29	0.00	1.00	3031
Trust in WHO	0.77	1.00	0.42	0.00	1.00	3037
Trust in Federal Government	0.66	1.00	0.47	0.00	1.00	3068
Trust in Angela Merkel	0.62	1.00	0.49	0.00	1.00	3064
Trust in Scientists	0.87	1.00	0.34	0.00	1.00	3041
Belief in Infecting Myself	4.10	4.00	1.27	1.00	7.00	3094
Belief in Infecting Others	3.91	4.00	1.29	1.00	7.00	3093
Belief Hospitalization Possible	3.28	3.00	1.30	1.00	7.00	3089
Belief Contacts Infect Themselves	4.60	5.00	1.42	1.00	7.00	3097
Belittle Threat of COVID-19	0.03	0.00	0.18	0.00	1.00	3685
Female	0.48	0.00	0.50	0.00	1.00	3685
Age: under 25	0.03	0.00	0.17	0.00	1.00	3685
Age: 26-60	0.67	1.00	0.47	0.00	1.00	3685
Age above 60	0.30	0.00	0.46	0.00	1.00	3685
Low Education	0.11	0.00	0.31	0.00	1.00	3685
Middle Education	0.31	0.00	0.46	0.00	1.00	3685
High Education	0.58	1.00	0.49	0.00	1.00	3685
1-Person Household	0.11	0.00	0.31	0.00	1.00	3685
2-Person Household	0.47	0.00	0.50	0.00	1.00	3685
3 or more Person in Household	0.42	0.00	0.49	0.00	1.00	3685
Employed	0.60	1.00	0.49	0.00	1.00	3102
Self-Employed	0.06	0.00	0.24	0.00	1.00	3102
Unemployed	0.01	0.00	0.12	0.00	1.00	3102
Out of Labor Force	0.32	0.00	0.47	0.00	1.00	3102

Notes: This table reports summary statistics of the individual-level data from the special COVID-19 questionnaire of the GESIS Panel Study (GESIS Panel Team, 2020).

Table A7: Mitigation Behavior and Trust in Institutions

	Dependent Variable: Mitigation Efforts						
	(1)	(2)	(3)	(4)	(5)	(6)	(7)
Trust Measure:	Local Health Office	Health Ministry	RKI	WHO	Federal Government	Angela Merkel	Scientists
Trust Measure	0.476***	0.570***	0.838***	0.528***	0.581***	0.575***	0.715***
	(0.066)	(0.065)	(0.119)	(0.069)	(0.059)	(0.056)	(0.096)
Female	0.266***	0.221***	0.256***	0.210***	0.249***	0.226***	0.280***
	(0.053)	(0.051)	(0.051)	(0.052)	(0.051)	(0.051)	(0.051)
Age: $26 - 60$	0.608***	0.627***	0.667***	0.659***	0.578***	0.582***	0.654***
	(0.219)	(0.211)	(0.209)	(0.207)	(0.204)	(0.203)	(0.205)
Age: > 60	0.732***	0.722***	0.764***	0.791***	0.658***	0.636***	0.775***
	(0.220)	(0.214)	(0.211)	(0.209)	(0.206)	(0.205)	(0.207)
Secondary Education	0.121	0.152	0.161	0.171*	0.130	0.126	0.125
	(0.104)	(0.100)	(0.098)	(0.101)	(0.100)	(0.100)	(0.101)
Tertiary Education	0.325***	0.310***	0.321***	0.328***	0.279***	0.256***	0.291***
	(0.099)	(0.096)	(0.095)	(0.097)	(0.096)	(0.096)	(0.097)
Persons in Household: 2	0.278***	0.304***	0.290***	0.307***	0.307***	0.309***	0.300***
	(0.097)	(0.093)	(0.091)	(0.094)	(0.092)	(0.092)	(0.092)
Persons in Household: ≥ 3	0.421***	0.457***	0.455***	0.452***	0.458***	0.466***	0.449***
	(0.102)	(0.098)	(0.097)	(0.099)	(0.097)	(0.097)	(0.097)
Self-Employed	-0.011	-0.007	0.009	-0.001	0.023	0.006	0.013
	(0.127)	(0.121)	(0.117)	(0.121)	(0.119)	(0.120)	(0.119)
Unemployed	-0.607*	-0.599**	-0.585**	-0.677**	-0.577**	-0.589**	-0.603**
	(0.317)	(0.298)	(0.290)	(0.309)	(0.294)	(0.292)	(0.298)
Out of Labor Force	-0.127*	-0.137*	-0.113	-0.123*	-0.126*	-0.099	-0.128*
	(0.074)	(0.071)	(0.072)	(0.071)	(0.071)	(0.070)	(0.072)
Outcome Mean	0.006	0.003	0.011	0.001	0.004	0.008	0.003
adj. R-Squared	0.049	0.058	0.058	0.052	0.064	0.065	0.057
Observations	2857	3052	3026	3031	3062	3058	3035

Notes: The table reports OLS regression results of a dummy variable *Mitigation Efforts* capturing COVID-19 mitigation behavior on a dummy variable *Trust Measure* indicating whether an individual trust the respective institution in column (1) to (7). The dependent variable *Mitigation Efforts* is the principal component of a series of questions regarding COVID-19 mitigation behavior (see Table A6 for further information). Control variables are listed. Robust standard errors are displayed in parentheses. Statistical significance is indicated by asterisks according to: * $p < 0.10$, ** $p < 0.05$, *** $p < 0.01$.

Table A8: Mitigation Behavior and Beliefs about COVID-19

	Dependent Variable: Mitigation Efforts				
	(1)	(2)	(3)	(4)	(5)
Belief Measure:	Infect Myself	Infect Others	Hospitalization	Contact Infected	Belittle COVID-19
Belief Measure	0.179***	0.116***	0.180***	0.155***	-0.768***
	(0.024)	(0.022)	(0.020)	(0.021)	(0.172)
Female	0.259***	0.255***	0.262***	0.281***	0.266***
	(0.051)	(0.051)	(0.051)	(0.051)	(0.051)
Age: $26 - 60$	0.656***	0.674***	0.543***	0.660***	0.648***
	(0.202)	(0.205)	(0.207)	(0.203)	(0.207)
Age: > 60	0.867***	0.840***	0.624***	0.899***	0.793***
	(0.204)	(0.207)	(0.208)	(0.205)	(0.208)
Secondary Education	0.136	0.155	0.185*	0.159	0.150
	(0.099)	(0.100)	(0.099)	(0.099)	(0.100)
Tertiary Education	0.278***	0.301***	0.385***	0.254***	0.308***
	(0.095)	(0.096)	(0.095)	(0.095)	(0.096)
Persons in Household: 2	0.281***	0.287***	0.283***	0.275***	0.279***
	(0.091)	(0.092)	(0.091)	(0.091)	(0.091)
Persons in Household: ≥ 3	0.404***	0.402***	0.440***	0.409***	0.421***
	(0.097)	(0.098)	(0.097)	(0.097)	(0.097)
Self-Employed	0.004	-0.001	-0.029	0.002	-0.019
	(0.120)	(0.122)	(0.119)	(0.118)	(0.119)
Unemployed	-0.504*	-0.595**	-0.684**	-0.567*	-0.559*
	(0.283)	(0.292)	(0.284)	(0.291)	(0.287)
Out of Labor Force	-0.082	-0.086	-0.158**	-0.106	-0.127*
	(0.070)	(0.070)	(0.071)	(0.070)	(0.070)
Outcome Mean	0.005	0.008	0.006	0.005	0.004
adj. R-Squared	0.051	0.038	0.053	0.050	0.038
Observations	3071	3071	3067	3074	3101

Notes: The table reports OLS regression results of a dummy variable *Mitigation Efforts* capturing COVID-19 mitigation behavior on a variable *Belief Measure* that captures individual beliefs about how likely it is to contract SARS-CoV-2, get hospitalized, infect others, or whether individuals belittle the threats posed by COVID-19. The dependent variable *Mitigation Efforts* is the principal component of a series of questions regarding COVID-19 mitigation behavior (see Table A6 for further information). Control variables are listed. Robust standard errors are displayed in parentheses. Statistical significance is indicated by asterisks according to: * $p < 0.10$, ** $p < 0.05$, *** $p < 0.01$.

Figure A2: SARS-CoV-2 Infection Rates in Germany

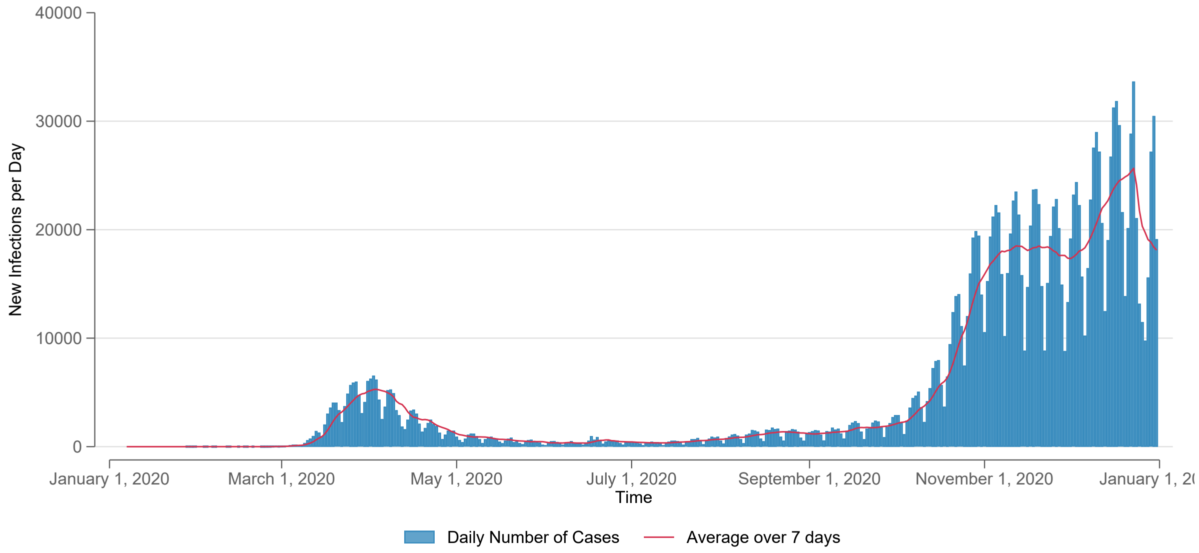

(a) Number of New Cases of SARS-CoV-2

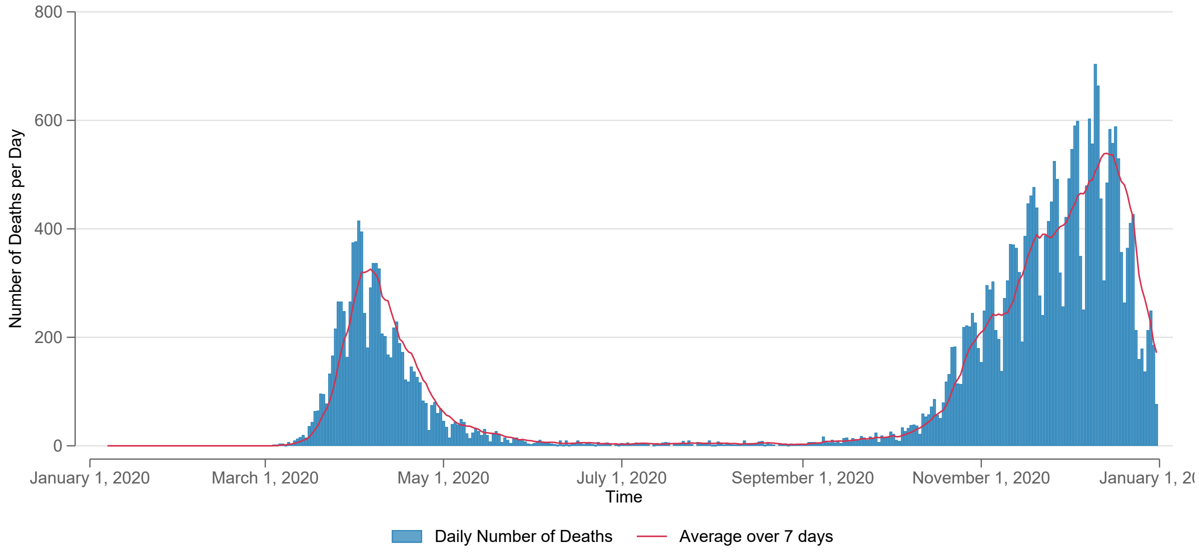

(b) Number of Deaths Associated with COVID-19

Notes: The figure shows the development of the number of SARS-CoV-2 cases and deaths related to COVID-19 in Germany during 2020.

Figure A3: Seven-days-incidence rates by treatment status

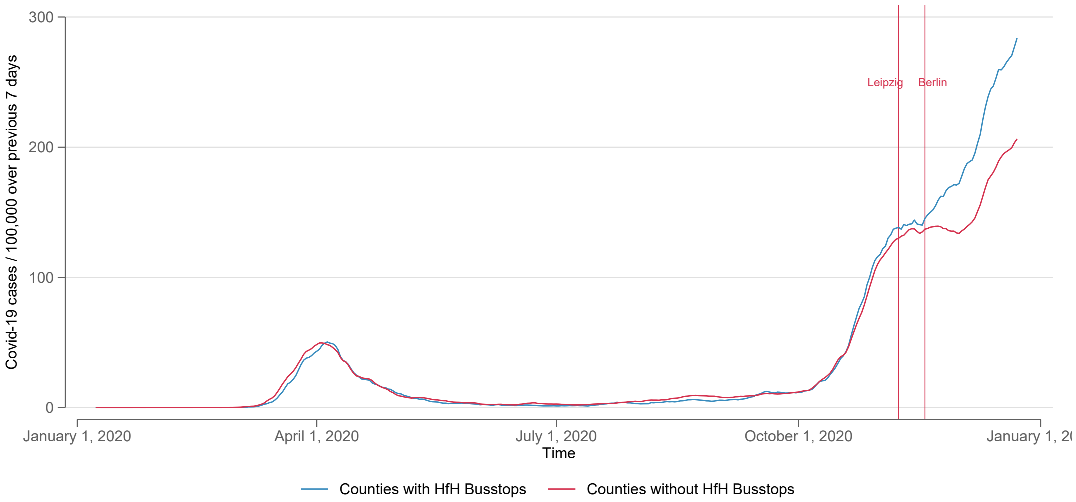

(a) Treated: HfH (<20,000)

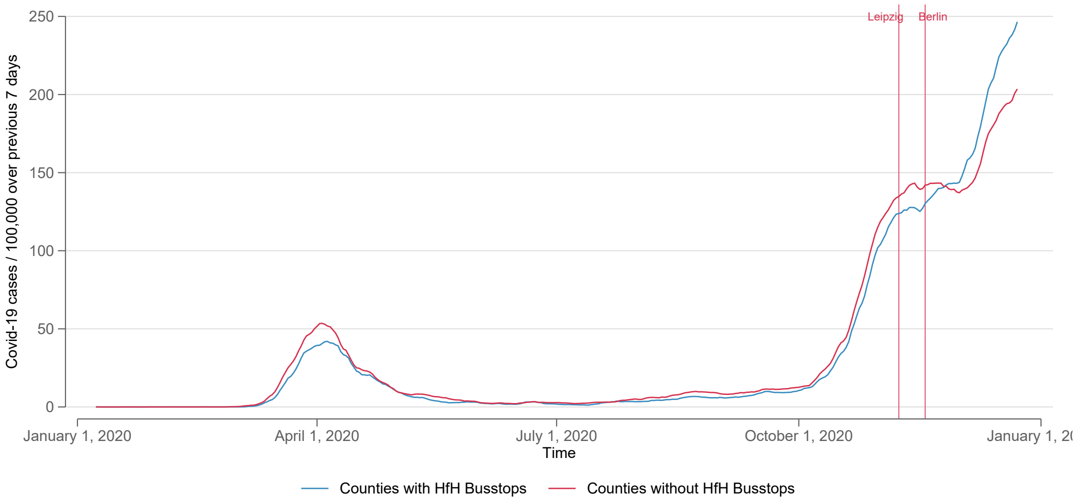

(b) Treated: HfH (<50,000)

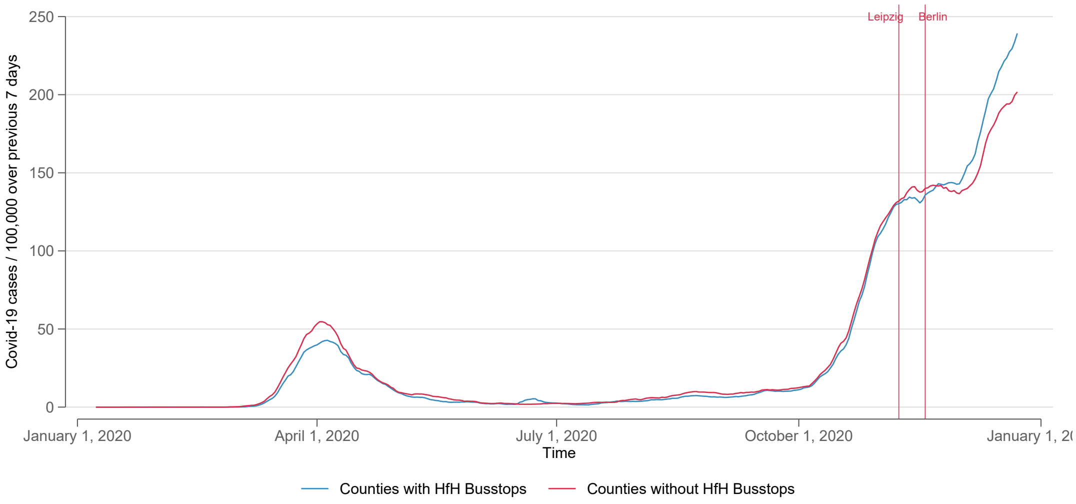

(c) Treated: HfH (all)

Notes: The figure shows the development of the seven-days-incidence rate during 2020 by treatment status.

Figure A4: Event Study Results with November 7 as Baseline Period

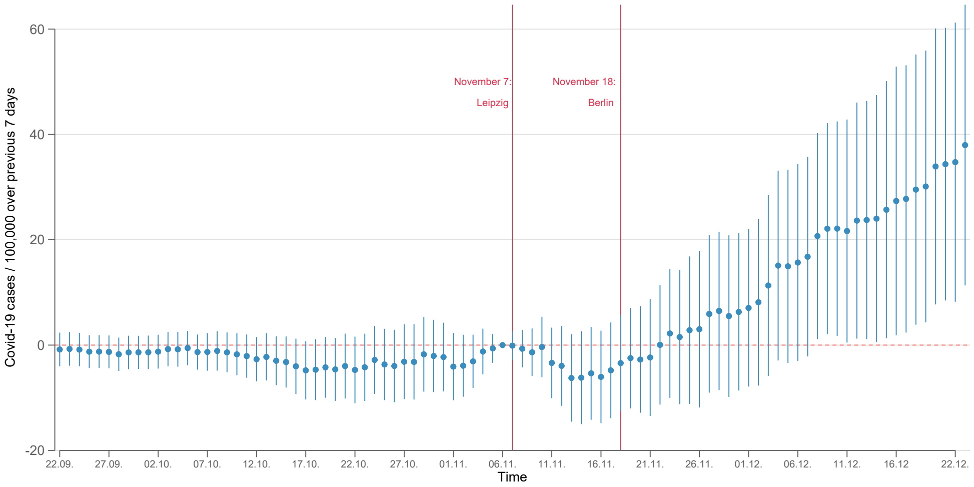

(a) Treated: Honk for Hope Stops

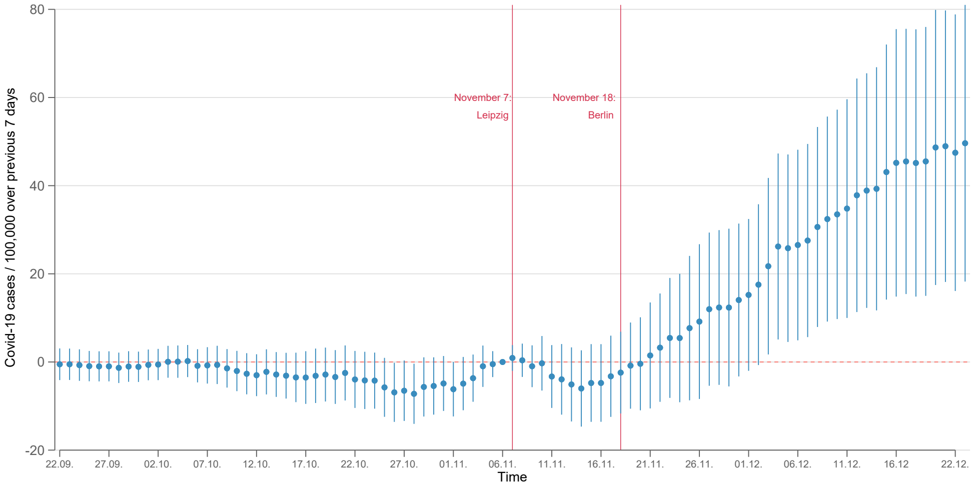

(b) Treated: Honk for Hope Stops (Cities Smaller than 50,000)

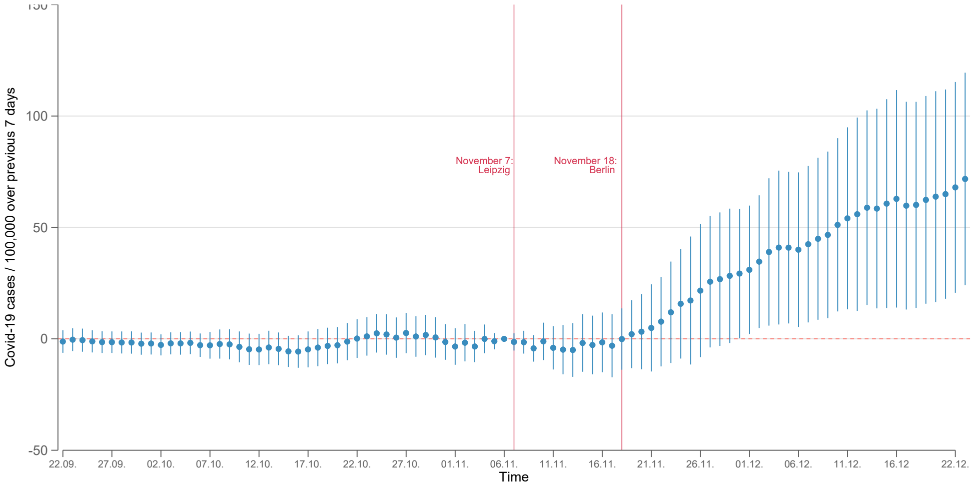

(c) Treated: Honk for Hope Stops (Cities Smaller than 20,000)

Notes: The figures plot the event study coefficients and their 95 percent confidence intervals on the effect of the Querdenken demonstrations in Leipzig and Berlin on the seven-days-incidence rates in German counties. The treated group are counties with Honk for Hope bus stops in general, in cities with fewer than 50,000 inhabitants, or in cities with fewer than 20,000 inhabitants. The sample is restricted to rural counties and a 46-day window around November 7. All models control for county and day fixed effects, as well as interactions between days and the incidence rate on November 7.

Figure A5: Robustness of the Event Study Results

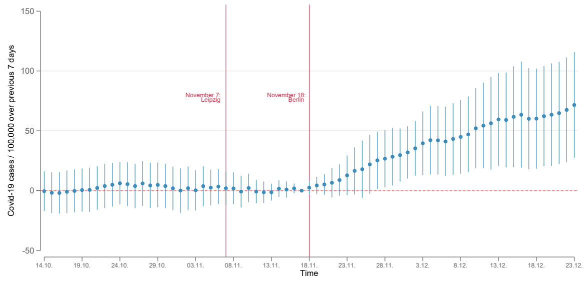

(a) Social Capital

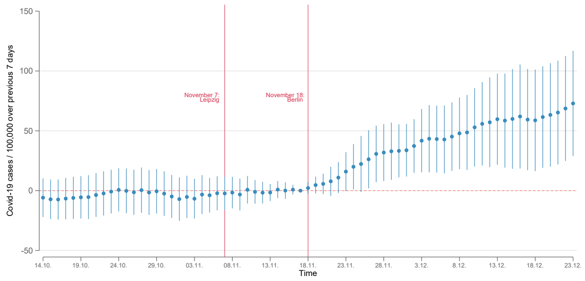

(b) FlixBus Stops (< 20,000)

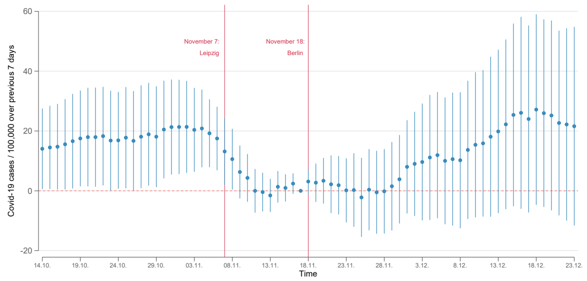

(c) Placebo: FlixBus Stops (< 20,000)

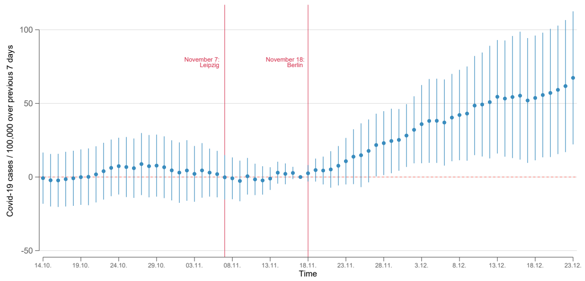

(d) Structural Factors

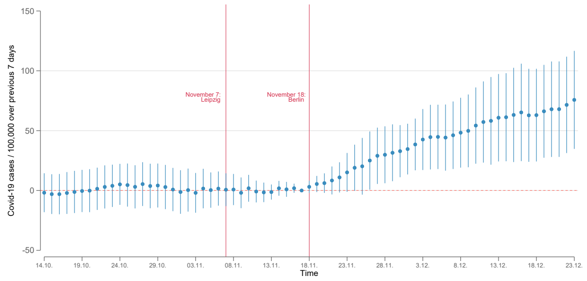

(e) Economic Factors

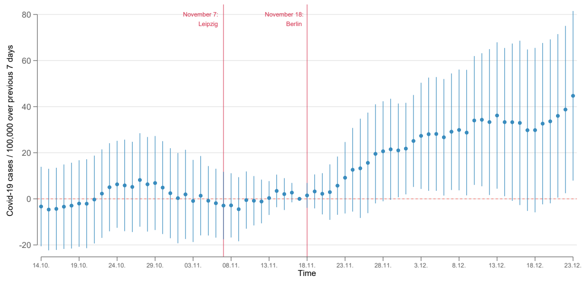

(f) All add. Controls

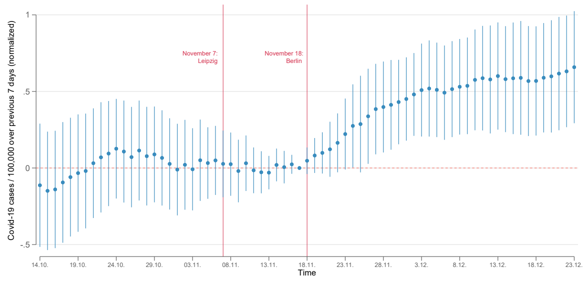

(g) Outcome: Normalized

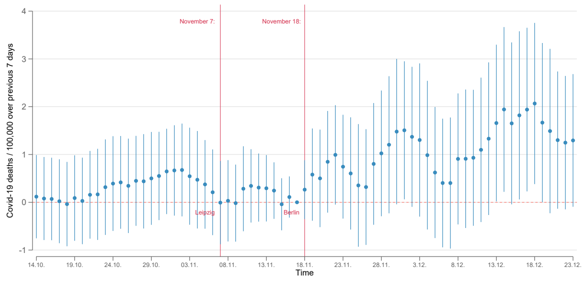

(h) Outcome: Seven-Days-Fatality Rate

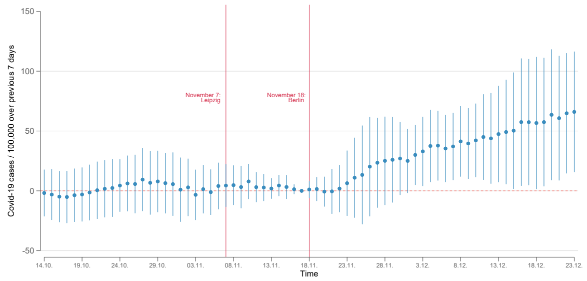

(i) Treatment: HfH to Leipzig

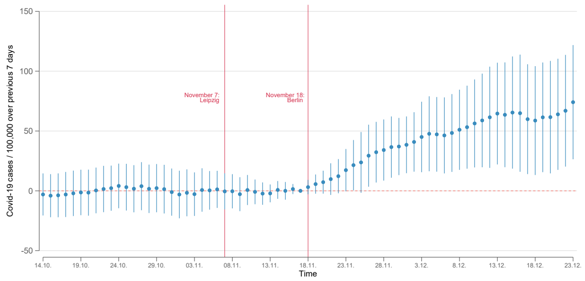

(j) Treatment: HfH to Berlin

Notes: The figures plot event study coefficients and their 95 percent confidence intervals for different robustness checks. As noted in the respective caption, X_c in equation (1) is extended by different control variables in (a)-(f); alternative outcomes are used in (g) and (h); and alternative treatment definitions are employed in (i) and (j).

Figure A6: Robustness: Drop Federal States I

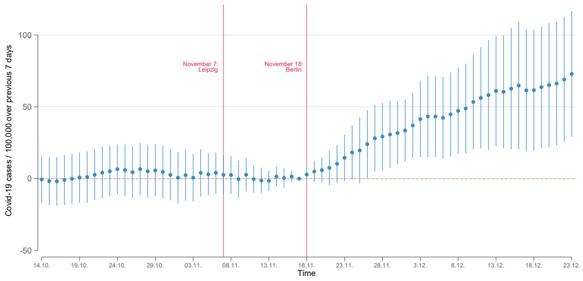

(a) Exclude Schleswig-Holstein

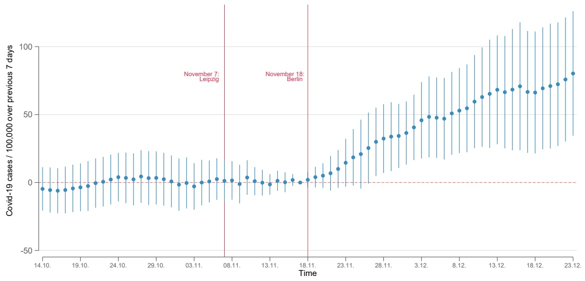

(b) Exclude Lower Saxony

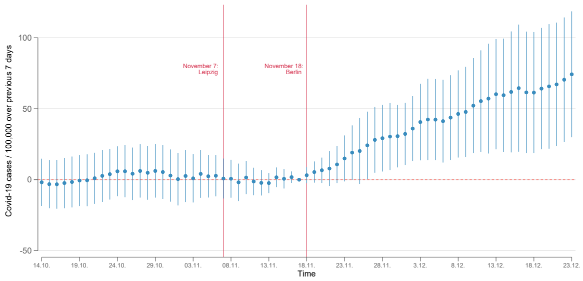

(c) Exclude North Rhine Westphalia

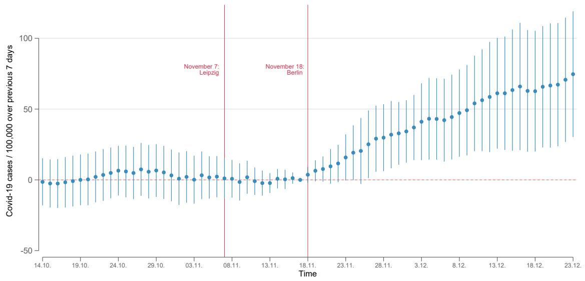

(d) Exclude Hesse

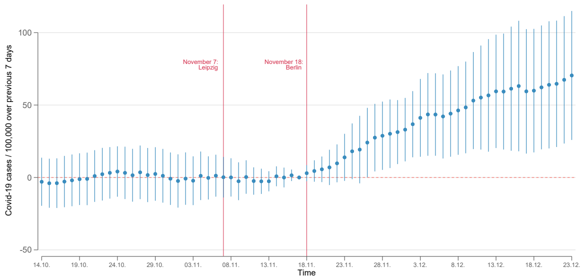

(e) Exclude Rhineland-Palatinate

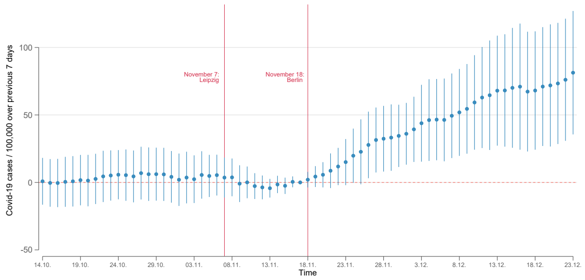

(f) Exclude Baden-Württemberg

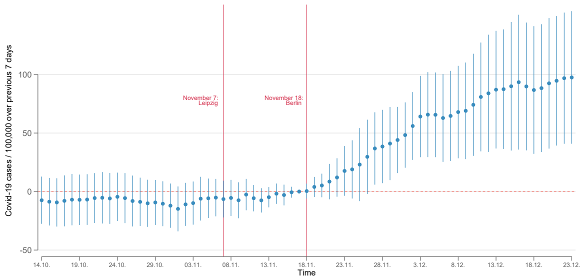

(g) Exclude Bavaria

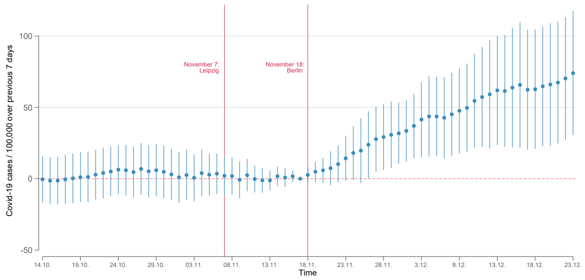

(h) Exclude Saarland

Notes: The figures plot the event study coefficients and their 95 percent confidence intervals on the effect of the Querdenken demonstrations in Leipzig and Berlin on the seven-days-incidence rates in German counties. Each subfigure excludes the state noted in the respective caption.

Figure A7: Robustness: Drop Federal States II

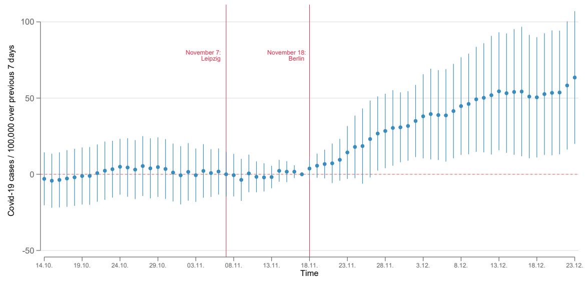

(a) Exclude Brandenburg

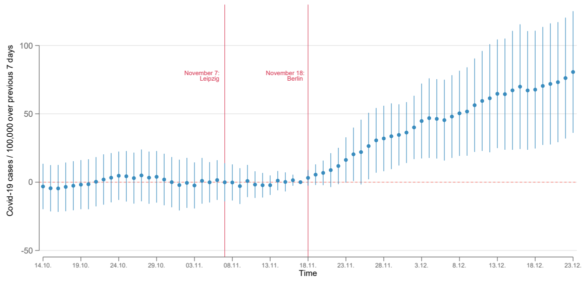

(b) Exclude Mecklenburg-West Pomerania

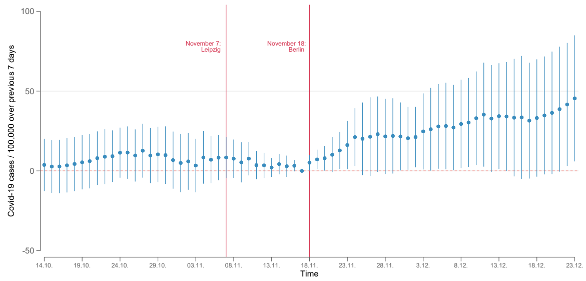

(c) Exclude Saxony

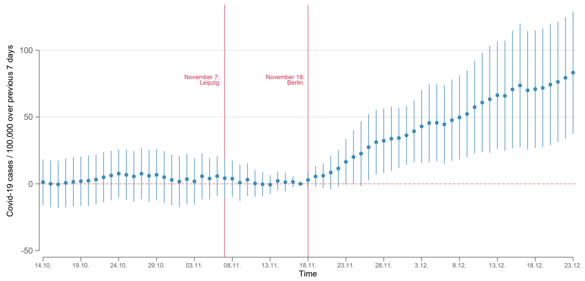

(d) Exclude Saxony Anhalt

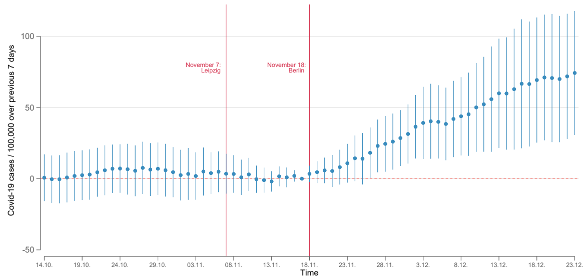

(e) Exclude Thuringia

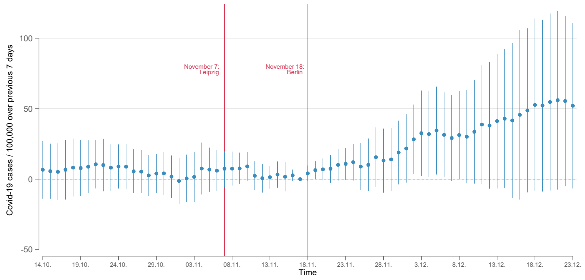

(f) Exclude Bavaria, Saxony, Thuringia

Notes: The figures plot the event study coefficients and their 95 percent confidence intervals on the effect of the Querdenken demonstrations in Leipzig and Berlin on the seven-days-incidence rates in German counties. Each subfigure excludes the state noted in the respective caption.

Figure A8: Robustness to Alternative Controls for Infection Dynamics

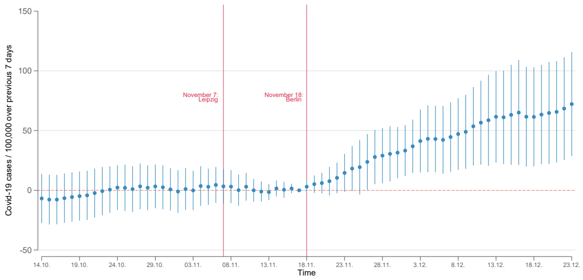

(a) Exclude Incidence 11/7

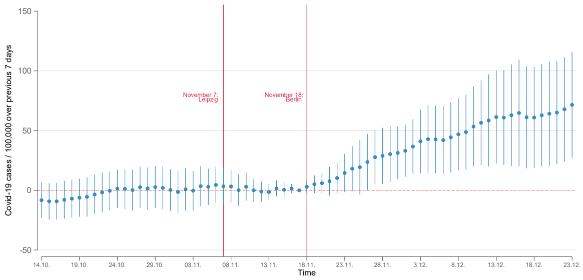

(b) Incidence 11/7 Quartiles

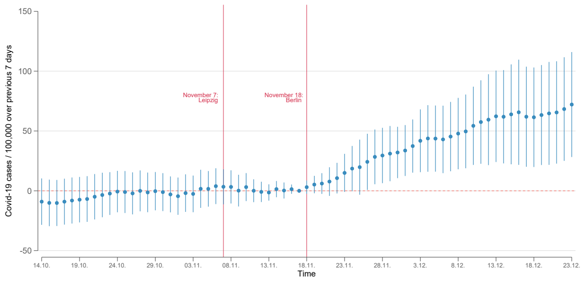

(c) Growth: $\frac{\text{Incidence } 11/7}{\text{Incidence } 10/31}$

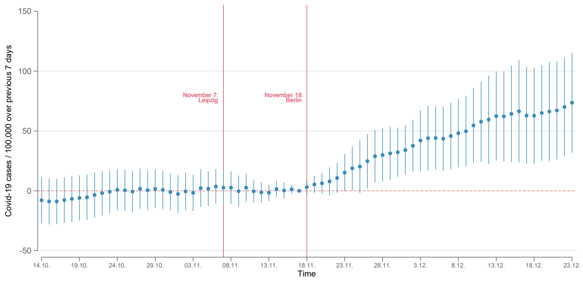

(d) Growth: $\frac{\text{Incidence } 11/7}{\text{Incidence } 10/31}$ Quartiles

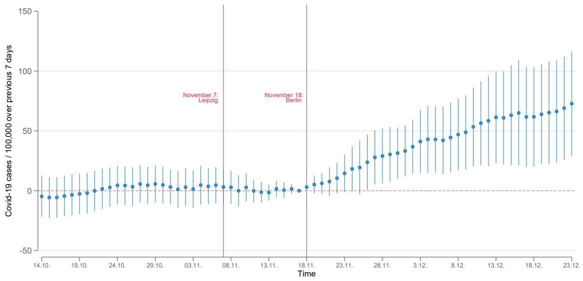

(e) Growth: Inc. 11/7 – Inc. 10/31

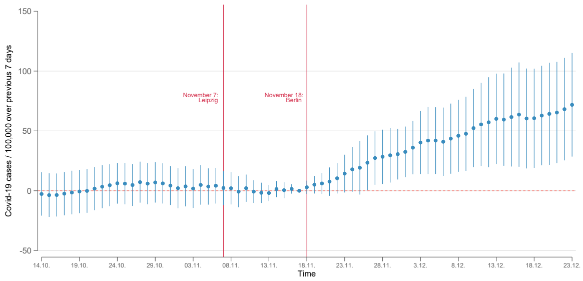

(f) Growth: Inc. 11/7 – Inc. 10/31 Quartiles

Notes: The figures plot the event study coefficients and their 95 percent confidence intervals on the effect of the Querdenken demonstrations in Leipzig and Berlin on the seven-days-incidence rates in German counties. Each subfigure includes a different set of control variables (interacted with time dummies) as described in the respective caption.

Figure A9: Heterogeneity of the Main Results

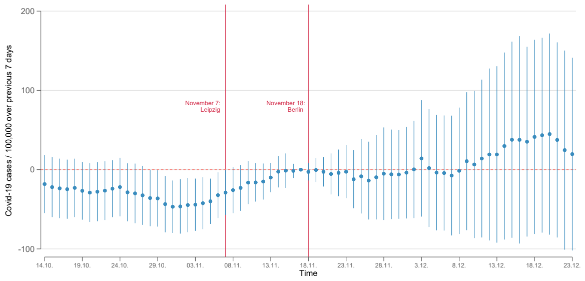

(a) Incidence Rate at November 7 – Interaction

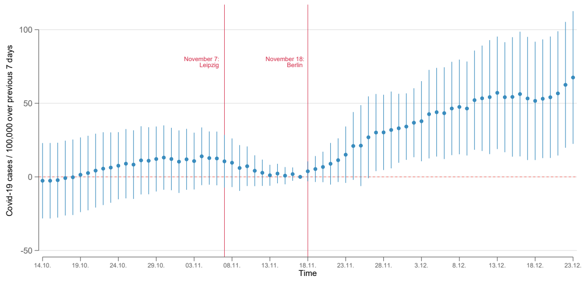

(b) Incidence Rate at November 7 – Main Estimate

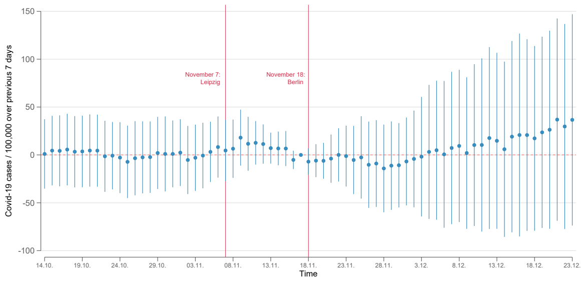

(c) Social Capital – Interaction

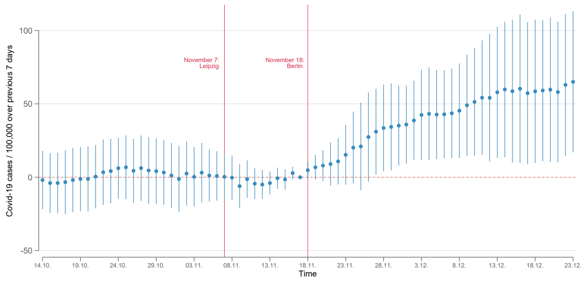

(d) Social Capital – Main Estimate

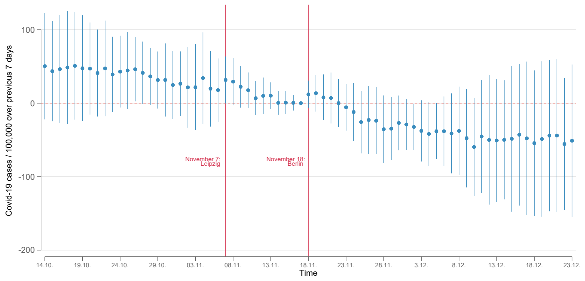

(e) Min. Distance to Leipzig / Berlin – Interaction

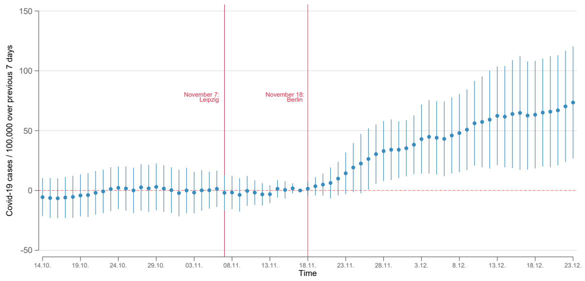

(f) Distance to Leipzig / Berlin – Main Estimate

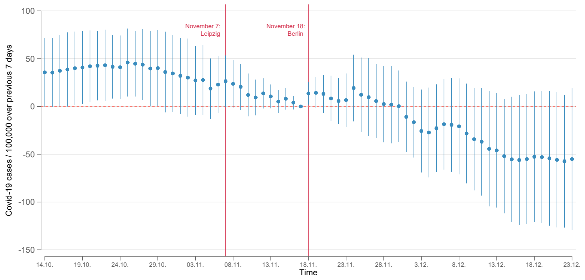

(g) GDP per capita – Interaction

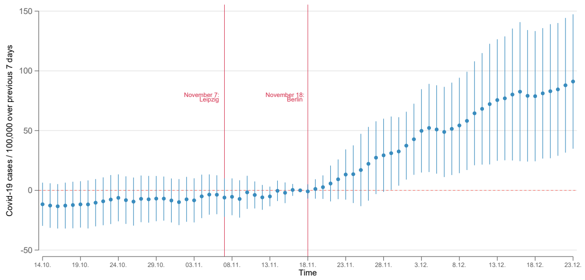

(h) GDP per capita – Main Estimate

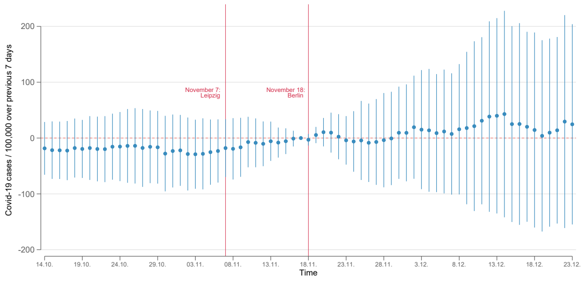

(i) Population density – Interaction

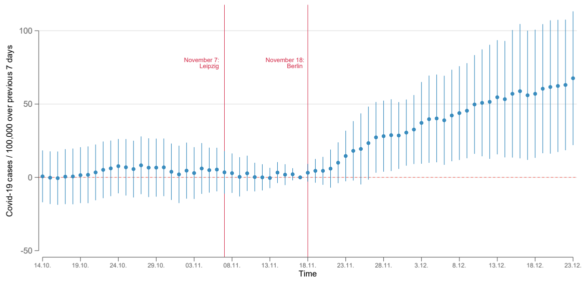

(j) Population density – Main Estimate

Notes: The figures plot event study coefficients and their 95 percent confidence intervals. The left column presents triple interactions of $D\{Bus_c\} \times D\{t = j\} \times X_c$ as well as the corresponding interaction terms $X_c \times D\{t = j\}$ for the respective covariate indicated in the caption. The right column shows the main effect $(D\{Bus_c\} \times D\{t = j\})$ for the specification on the left.

Bibliography

GESIS Panel Team (2020). GESIS Panel Special Survey on the Coronavirus SARS-CoV-2 Outbreak in Germany. GESIS Datenarchiv, Köln. ZA5667 Datenfile Version 1.1.0, https://doi.org/10.4232/1.13520.

Rieck, T., M. Feig, A. Siedler, and O. Wichmann (2018). Aktuelles aus der KV-Impfsurveillance – Impfquoten ausgewählter Schutzimpfungen in Deutschland. *Epidemiologisches Bulletin* (1), 1–14.
